# Highly Combinatorial Genetic Interaction Analysis Reveals a Multi-Drug Transporter Influence Network

**DOI:** 10.1016/j.cels.2019.09.009

**Published:** 2019-10-23

**Authors:** Albi Celaj, Marinella Gebbia, Louai Musa, Atina G. Cote, Jamie Snider, Victoria Wong, Minjeong Ko, Tiffany Fong, Paul Bansal, Joseph C. Mellor, Gireesh Seesankar, Maria Nguyen, Shijie Zhou, Liangxi Wang, Nishka Kishore, Igor Stagljar, Yo Suzuki, Nozomu Yachie, Frederick P. Roth

**Affiliations:** 1 Donnelly Centre, University of Toronto, Toronto, ON M5S 3E1, Canada; 2 Lunenfeld-Tanenbaum Research Institute, Mount Sinai Hospital, Toronto, ON M5G 1X5, Canada; 3 Department of Molecular Genetics, University of Toronto, Toronto, ON M5S 1A8, Canada; 4 Department of Computer Science, University of Toronto, Toronto, ON M5T 3A1, Canada; 5 Department of Biochemistry, University of Toronto, Toronto, ON M5S 1A8, Canada; 6 Mediterranean Institute for Life Sciences, Split 21 000, Croatia; 7 Department of Biological Chemistry and Molecular Pharmacology, Harvard Medical School, Boston, MA 02115, USA; 8 Synthetic Biology Division, Research Center for Advanced Science and Technology, University of Tokyo, Tokyo 153-8904, Japan; 9 Department of Biological Sciences, School of Science, University of Tokyo, Tokyo 113-0033, Japan; 10 Institute for Advanced Biosciences, Keio University, Yamagata 997-0035, Japan; 11 PRESTO, Japan Science and Technology Agency, Tokyo 153-8904, Japan; 12 Lead Contact

## Abstract

Many traits are complex, depending non-additively on variant combinations. Even in model systems, such as the yeast *S. cerevisiae*, carrying out the high-order variant-combination testing needed to dissect complex traits remains a daunting challenge. Here, we describe “*X*-gene” genetic analysis (XGA), a strategy for engineering and profiling highly combinatorial gene perturbations. We demonstrate XGA on yeast ABC transporters by engineering 5,353 strains, each deleted for a random subset of 16 transporters, and profiling each strain’s resistance to 16 compounds. XGA yielded 85,648 genotype-to-resistance observations, revealing high-order genetic interactions for 13 of the 16 transporters studied. Neural networks yielded intuitive functional models and guided exploration of fluconazole resistance, which was influenced non-additively by five genes. Together, our results showed that highly combinatorial genetic perturbation can functionally dissect complex traits, supporting pursuit of analogous strategies in human cells and other model systems.

## In Brief

Celaj et al. introduce “*X*-gene” genetic analysis (XGA), a strategy for modeling complex systems by engineering and profiling highly combinatorial genetic perturbations. They apply XGA to 16 yeast ABC transporters, revealing many high-order genetic interactions. Neural network models yielded intuitive functional models and illuminated an ABC transporter influence network, supporting application of XGA to other organisms and processes.

## Graphical Abstract

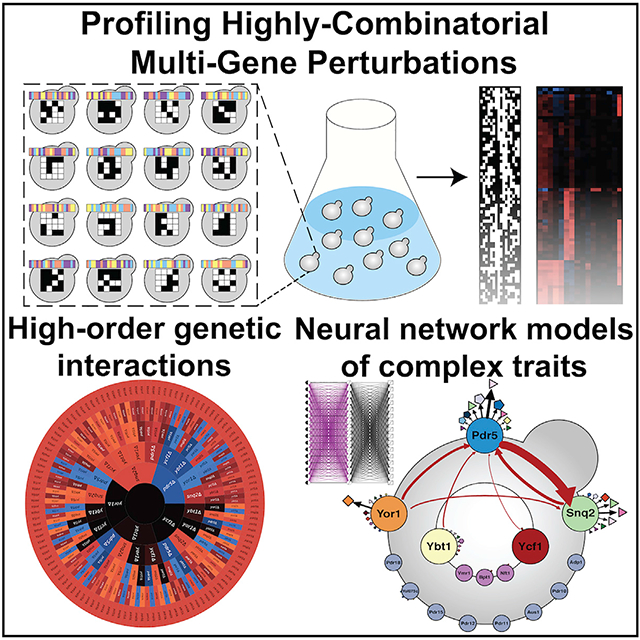

## INTRODUCTION

Genes often encode interdependent and functionally overlapping molecular systems, such that combinations of genetic variants can yield surprising phenotypes (Hartman et al., 2001). This phenomenon defines genetic interactions and gives rise to complex traits that cannot be understood by single-gene perturbations. Model eukaryotes, including the yeast *S. cerevisiae* and cultured human cells, have been an important testbed for understanding complex traits. Observing genetic interactions between pairs of genes, e.g., using synthetic genetic array analysis (SGA), has systematically uncovered functional relationships in yeast (Costanzo et al., 2016) and human cells (Horlbeck et al., 2018; Shen and Ideker, 2018), improving our understanding of gene function (Costanzo et al., 2016) and order-of-action in biological pathways (St Onge et al., 2007).

Genetic interactions with higher complexity, e.g., three-gene perturbations yielding phenotypes that are unexpected given the corresponding one- and two-gene perturbation phenotypes, can reveal additional important functions (Haber et al., 2013; Kuzmin et al., 2018). Indeed, three-gene interactions are expected to outnumber two-gene interactions by 100-fold (Kuzmin et al., 2018). Beyond three-gene interactions, higher-order gene variant combinations have yielded interesting effects—e.g., involving four (Takahashi and Yamanaka, 2006), five (Taylor and Ehrenreich, 2014), seven (Beh et al., 2001), and over twenty genes (Wieczorke et al., 1999). Systematic maps of higher-order interactions between variants at a single locus have been used to understand several diverse processes (Baeza-Centurion et al., 2019; Domingo et al., 2018; Sarkisyan et al., 2016). However, higher-order interactions between variants in different genes have remained poorly characterized, limiting functional understanding of complex multi-gene dependencies.

To systematically investigate complex genetic dependencies beyond one- and two-gene combinatorial analysis, we developed an “*X*-gene” genetic analysis (XGA) strategy that uses many combinations of engineered multi-gene perturbations to profile and interpret higher-order genetic interactions. We demonstrate XGA on *S. cerevisiae* ABC transporters, which are involved in cellular efflux of small molecules (Paumi et al., 2009) and for which several informative multi-knockout phenotypes have been reported (Khakhina et al., 2015; Kolaczkowska et al., 2008; Suzuki et al., 2011). More specifically, we apply XGA systematically to the entire set of 16 yeast ABC transporters that have been implicated in multi-drug resistance. By revealing a multi-knockout genetic landscape for 16 bioactive compounds (“drugs”), XGA uncovered many drug-dependent high-order genetic interactions involving as many as five genes. A neural network trained on XGA data provided an intuitive genotype-to-phenotype model and functional insights into this system of ABC transporters. Taken together, our results show that XGA can systematically uncover high-order genetic relationships and use them to model mechanism. These results support the potential power of analogous highly combinatorial perturbation strategies in human cells to functionally dissect other complex traits and associated molecular systems.

## RESULTS

### Cross-Based XGA: A Scheme for Generating and Characterizing Combinatorially Complex Mutants

**Figure 1.**
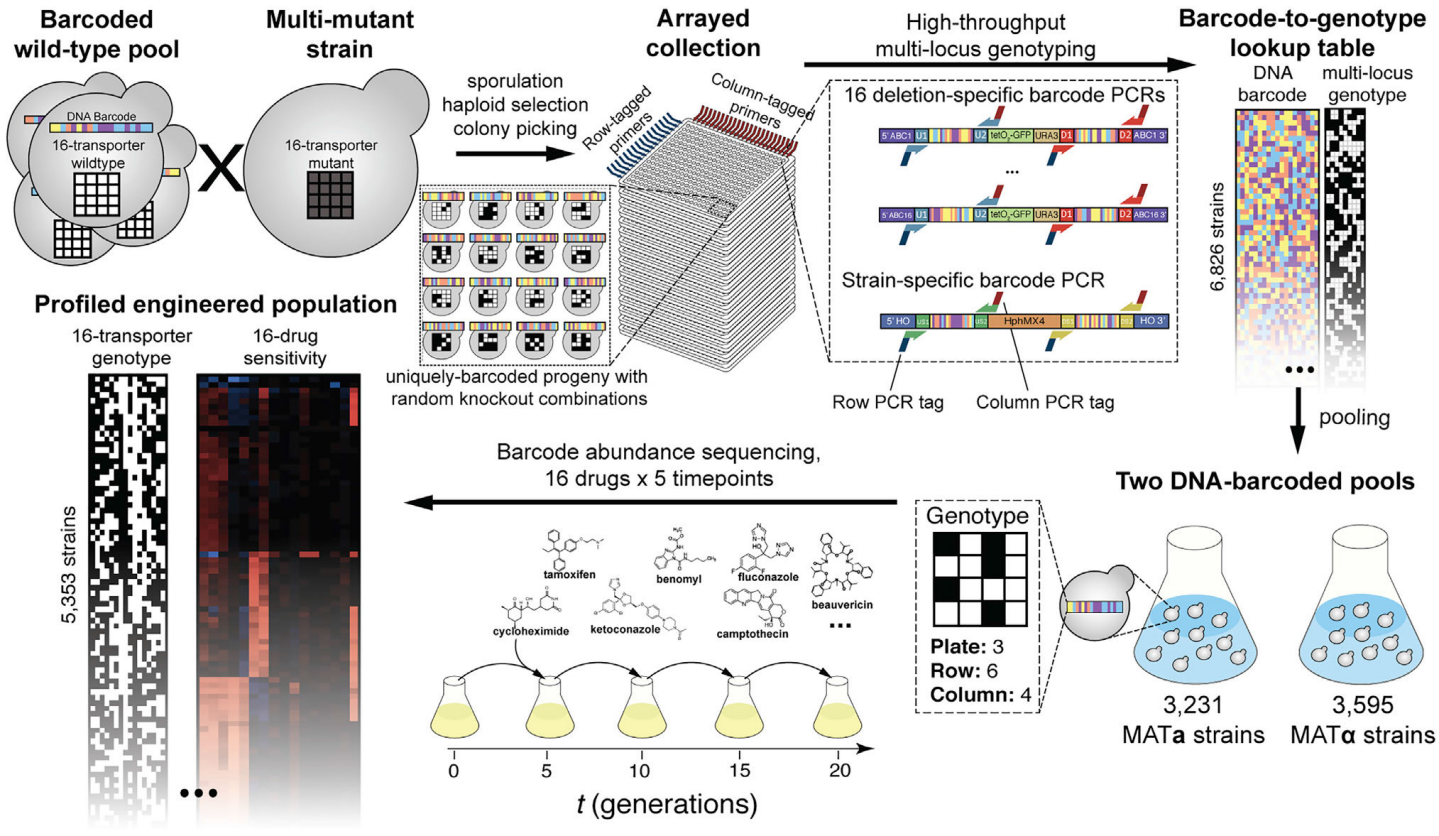
Overview of a Cross-Based Implementation of XGA A population is engineered by mating a barcoded pool of wild-type cells with a multi-mutant strain (here, the ABC-16 strain bearing 16 ABC transporter knockouts). Each haploid progeny strain inherits a unique DNA barcode and a random combination of knockout (black) and wild-type (white) alleles. Progeny are picked from single colonies and arrayed in 384-well plates. An *en masse* tag-based PCR indexing strategy associates the genotype of each strain to a DNA barcode. Strains are pooled by mating type. Pools are grown in specific environments (here in 16 drugs and a DMSO solvent control). High-throughput sequencing of strain-specific DNA barcodes at multiple time points reconstructs the resistance of each strain to each drug. See also Figure S1.

Here, we describe a variant of the XGA strategy that generates an “engineered population” by crossing a wild-type and a multi-knockout strain (Figure 1). We then show the results for this strategy as applied to 16 yeast ABC transporters. Briefly, targeted polygenic variation is engineered into a parental strain, such that a genetic cross yields a population in which only the engineered variation is segregating.

As recently reviewed (Kebschull and Zador, 2018), many individual strains can be tracked in a complex heterogenous population using DNA barcodes. We therefore introduced a complex pool of random barcodes into a haploid parental strain (which was wild type for all ABC transporter genes of interest in this study), as described previously (Díaz-Mejía et al., 2018) (Figures S1A and S1B). We crossed this barcoded wild-type pool *en masse* to a previously generated “ABC-16-strain”, which bears knockouts for all 16 of the ABC transporters that have been implicated in multi-drug resistance (Suzuki et al., 2011). The ABC-16 strain contained all markers necessary to perform mating, sporulation, and selection for haploid cells, while the barcoded wild-type parent provided the marker necessary to select for cells carrying a barcoded *HO* locus (Figure S1B). After mating, sporulation, and selection for barcoded haploid progeny of the cross, we used automated colony picking to isolate an arrayed collection of 5,760 MAT**a** and 5,760 MAT**α** segregants in 384-well plates. This step generated an engineered population in which each individual haploid strain bears a random subset of knockout alleles for the target set of 16 ABC transporters.

For each strain in this arrayed population, we determined the genotype at all 16 knockout loci and identified the barcode. To genotype, we exploited the fact that each knockout locus in the ABC-16 strain was derived from a YKO yeast deletion strain (Giaever et al., 2002; Suzuki et al., 2011) and is therefore flanked by a deletion-identifying barcode. We adapted the previously described row-column-plate PCR (RCP-PCR) strategy (Yachie et al., 2016), in which barcodes in each segregant are amplified together with additional PCR-introduced index tags that identify the plate, row, and column of origin for each amplification product (Data S1; Figure 1). Thus, a single sequencing experiment revealed both the strain-specific tracking barcode at the *HO* locus and the identity of every gene deleted in each segregant at each plate location (Data S2; Figure 1).

Two independent methods estimated the overall per-locus genotyping accuracy to be from 93.2% to 93.8% (Figures S1C and S1D). Based on correlation analysis of the genotyping data, all genes were either unlinked or weakly linked except for *BPT1* and *YBT1* (Figure S1E; *r* = 0.49), which are separated by 70.1 kb on chromosome XII. Considering only those strains with both high-quality genotyping data and at least one unique tracking barcode, our engineered strain population included 6,826 uniquely barcoded and genotyped strains, encompassing 6,087 unique genotypes. These strains were grouped by mating type to yield one pool of 3,231 MAT**a** strains and another pool of 3,595 MAT**α** strains.

To profile each strain’s resistance or sensitivity to 16 different bioactive compounds (“drugs;” Data S3), we grew the strain pools competitively in each drug and in a solvent (DMSO) control condition. The drugs tested included seven anti-cancer drugs, four azole antifungals, and five other compounds that are useful chemical probes or are potential anti-microbials (Data S3). Ten of these compounds had an established phenotype for knockouts of *SNQ2*, *PDR5*, or *YOR1* (Data S3). We used high-throughput strain barcode sequencing at five time points (corresponding to 0, 5, 10, 15, and 20 generations of overall pool growth, Figure 1) to estimate growth rate and resistance for each strain in each drug (Data S4; STAR Methods). We performed additional filtering steps, limiting analysis to strains that were well-represented in the pre-selection pool (≥30 barcode counts at t = 0 in the solvent control), which captured 5,790 (85%) of 6,826 strains. We further excluded all 437 strains exhibiting a strong baseline growth defect (i.e., showing <70% of the median baseline growth rate). In total, drug resistance was calculated for each of 2,367 MAT**a** and 2,986 MAT**α** strains, for each of the 16 drugs (Data S4).

### Grouped Combinatorial Profiles Illustrate a Complex and Drug-Dependent Genetic Landscape

**Figure 2.**
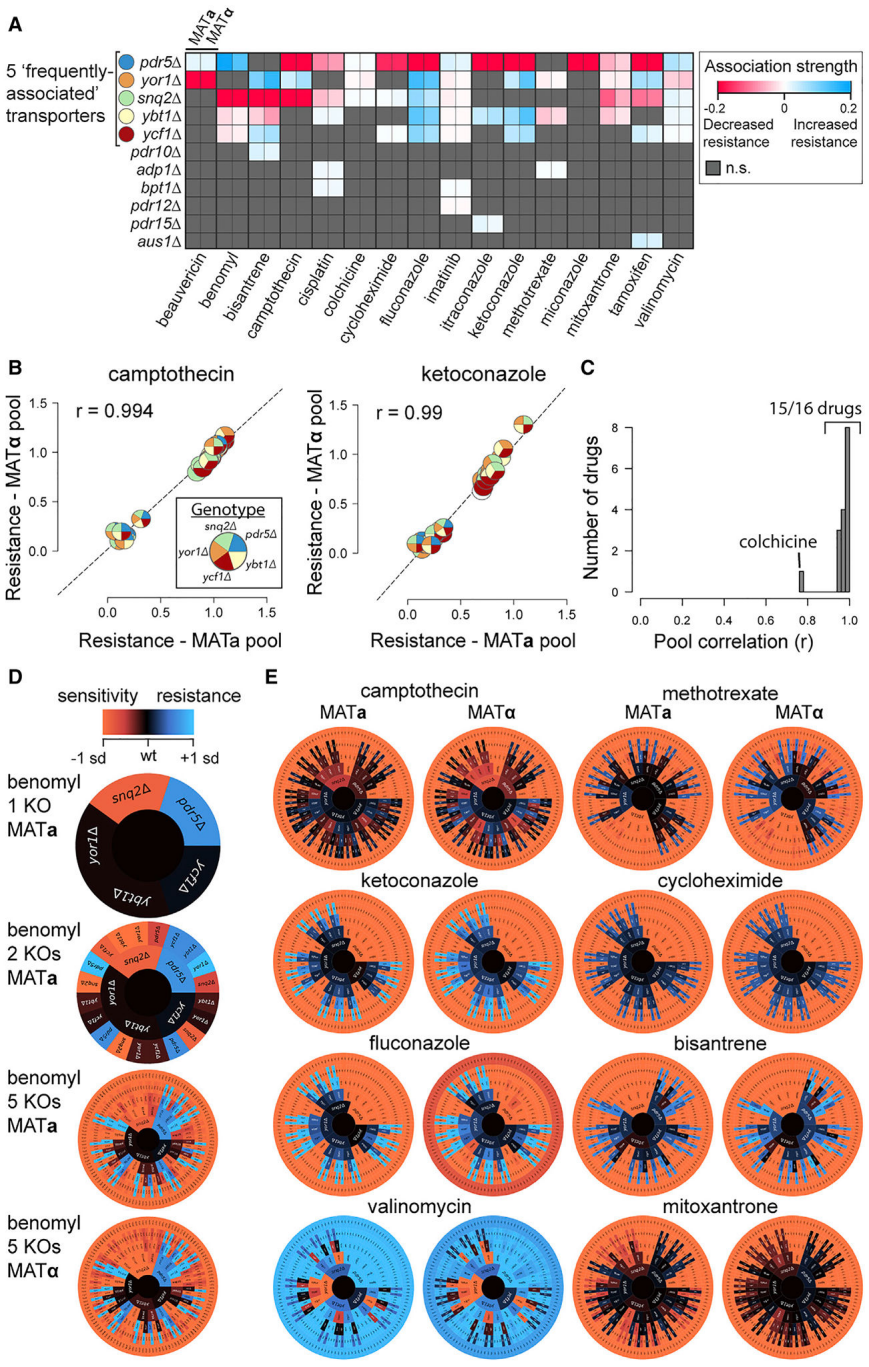
Illustrating a Reproducible Five-Gene Resistance Landscape (A) Knockout effects on drug resistance averaged over all genetic backgrounds. A linear model identified significant single-gene marginal effects (p < 0.05 after adjusting for multiple testing). Those found in both MAT**a** and MAT**α** pools are shown. Five transporter genes were identified as being frequently associated with drug resistance traits. See also Figure S2A. (B) Comparison of five-gene camptothecin and ketoconazole resistance profiles between MAT**a** and MAT**α** pools. Mean resistance was calculated for strains belonging to each five-gene genotype group, averaging over other genotypes. See also Figure S2B. (C) Five-gene resistance profiles were highly reproducible between MAT**a** and MAT**α** pools for 15 of 16 drugs tested. (D) A five-gene “XGA wheel” of benomyl resistance. Strains were grouped as in Figure 2B. Color at the center represents the mean resistance of the five-gene wild-type group, while radial segments extending outwards represent the mean resistances of strains grouped by the indicated series of cumulatively added knockout alleles, relative to the five-gene wild type. Extensions to 1, 2, and 5 total knockouts are illustrated. Each section adds *ycf1Δ*, *ybt1Δ*, *yor1Δ*, *snq2Δ*, and *pdr5Δ* in clockwise order, excluding previously-added knockouts. The color scale for each pool ranges between one standard deviation below and above the observed drug resistance values. (E) XGA wheels for 8 drugs, as defined in Figure 2C. See also Figure S3.

For an initial analysis, we identified and quantitatively modeled associations between individual ABC transporter knockouts and drug resistance phenotypes using a generalized linear model (STAR Methods). We found 62 associations between individual knockouts and drug resistance that were reproducible in both MAT**a** and MAT**α** pools (Figure 2A). Most (58/62) of these associations involved five “frequently associated” ABC transporters—*snq2Δ*, *pdr5Δ*, *yor1Δ*, *ycf1Δ*, and *ybt1Δ* (Figure 2A).

For these five frequently associated transporters, we detected 16 of 18 previously reported associations between drug resistance and individual knockouts, while revealing 40 additional associations (Datas S3 and S5). For example, we detected 18 drug resistance associations involving the vacuolar ABC transporters *YCF1* and *YBT1*, none of which had been previously reported (Figure 2A; Data S5). We also found 4 associations between knockouts and growth rate in the DMSO control condition (Figure S2A; Data S5). Of these four associations with baseline growth, only *yor1Δ* had an appreciable effect (7%–15% decrease), while the other three baseline growth effects were quite weak (<2% decrease; Figure S2A; Data S5).

Again considering only the five frequently associated transporters, we calculated the average resistance over strain groups that correspond to one of the 32 (2^5^) possible combinatorial genotypes (ignoring genotype outside the five frequently associated genes). The resistance profiles for these strain groups showed high reproducibility when calculated separately for MAT**a** and MAT**α** pools (Figure S2B). For example, camptothecin and ketoconazole each showed correlations of *r* ≥ 0.99 (Figure 2B). Colchicine showed the least correlation between biological replicates (*r* = 0.77) but also the lowest absolute error (Figure S2B). This suggests that the lower reproducibility of colchicine stems from a lesser dependence of resistance on the genotypes tested, as the resistance of the five-gene groups ranged only from 0.98 to 1.02 (Figure S2B).

The five-gene resistance profiles could be used to provide a visual overview of multi-knockout resistance in each drug. We created an “XGA wheel” for each drug, representing the consequences of knocking out increasingly many ABC transporters as paths leading outward from the central wild-type genotype (Figures 2D and 2E). As expected, XGA wheels were visually similar between independent MAT**a** and MAT**α** populations for many drugs (Figures 2E and S3). These landscapes also highlighted high-order combinations of ABC transporters, which, when deleted, led to greater drug resistance (Figures 2E and S3). Given high reproducibility, MAT**a** and MAT**α** data were merged for subsequent analyses, except where noted.

**Figure 3.**
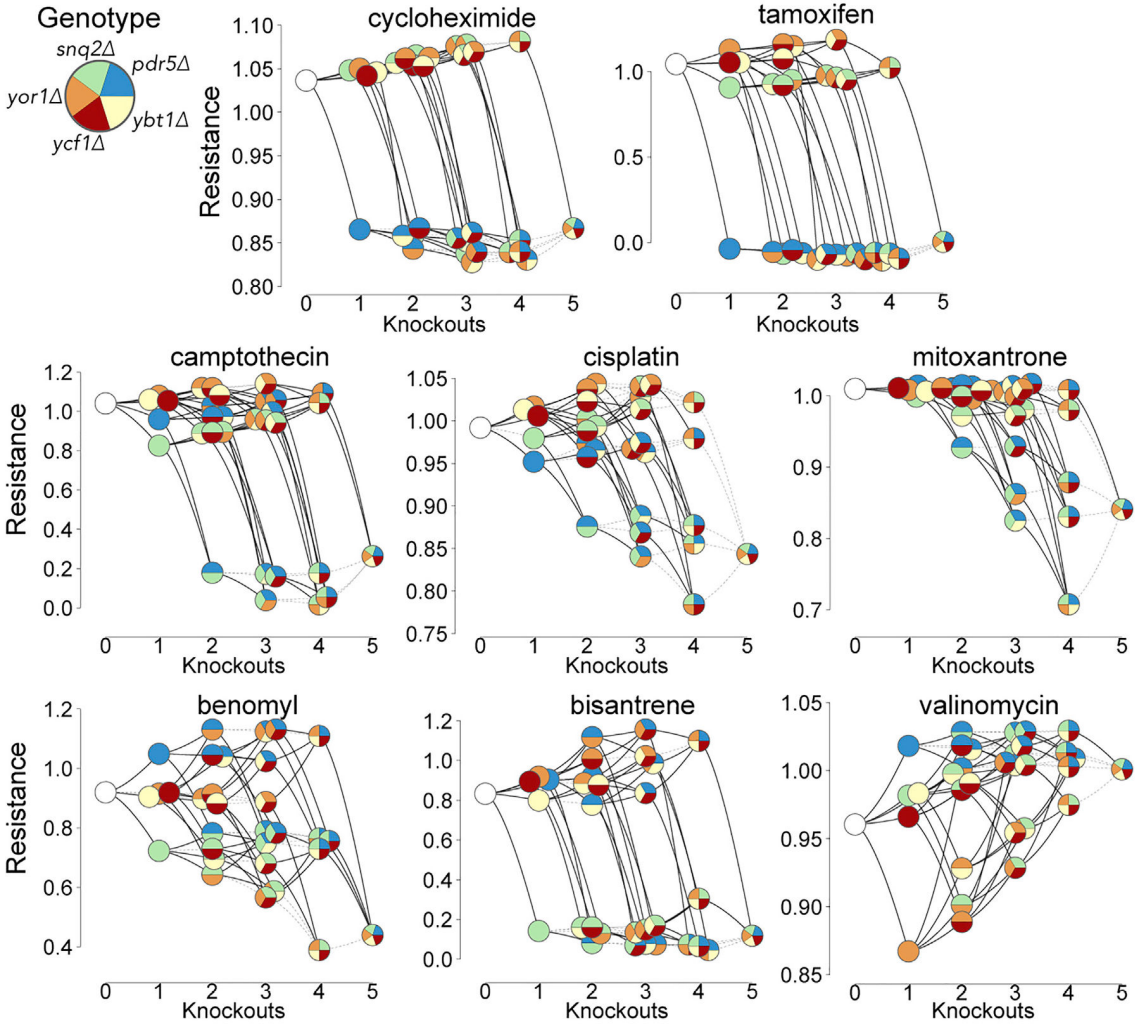
Five-Gene Knockout Landscapes Illustrate Complex Drug Resistance Effects Resistance landscapes of strain groups defined by genotypes at five frequently associated transporters are shown for eight drugs. Groups differing by a single additional knockout are connected by lines. Solid lines indicate significant differences in resistance (multiple-testing-adjusted p < 0.05, Mann-Whitney *U* test), otherwise dashed lines are used. See also Figure S4.

To analyze the five-gene combinatorial resistance profiles in more detail, we visualized them as fitness landscapes (Ferretti et al., 2018) (Figures 3 and S4). For some drugs, these landscapes illustrated a clear sensitivity effect from knocking out only one transporter—e.g., *pdr5Δ* for cycloheximide and tamoxifen (Figure 3). In other drugs, we saw increased sensitivity resulting from knocking multiple transporters—e.g., the set {*snq2Δ*, *pdr5Δ*} under camptothecin and the set {*snq2Δ*, *pdr5Δ*, *ybt1Δ*, *yor1Δ*} under mitoxantrone (Figure 3). These sensitivity patterns are consistent with a relatively simple scenario in which one or more transporters can efflux a given drug.

For other drugs, the fitness landscapes showed multi-knockout patterns conveying both drug resistance and sensitivity. In benomyl, for example, we observed not only the expected sensitivity in knockouts of the known primary efflux pump *snq2Δ* (20% decreased resistance, *p* = 1.4e-95; Mann-Whitney *U* test) but also 13% increased resistance in *pdr5Δ* knockouts (p = 1.3e-41) and further resistance in the *pdr5Δ yor1Δ* double-mutant (21% increased resistance; p = 1.3e-72). All of these effects had been previously reported (Kolaczkowska et al., 2008; Snider et al., 2013) and have been explained by *SNQ2*-mediated resistance that increases upon deleting *pdr5Δ* and *yor1Δ*. Consistent with this explanation, the deletion-dependent benomyl resistance effects we observed were more modest in a *snq2Δ* background (Figure 3). A similar landscape was found in bisantrene, which also showed a strong *snq2Δ* sensitivity effect (Figure 3). In valinomycin, only *yor1Δ* showed sensitivity, whereas knocking out *pdr5Δ*, *snq2Δ*, *ybt1Δ*, and *ycf1Δ* (individually or in any combination) led to greater resistance (Figure 3).

### XGA Reveals Many Drug-Dependent High-Order Genetic Interactions

**Figure 4.**
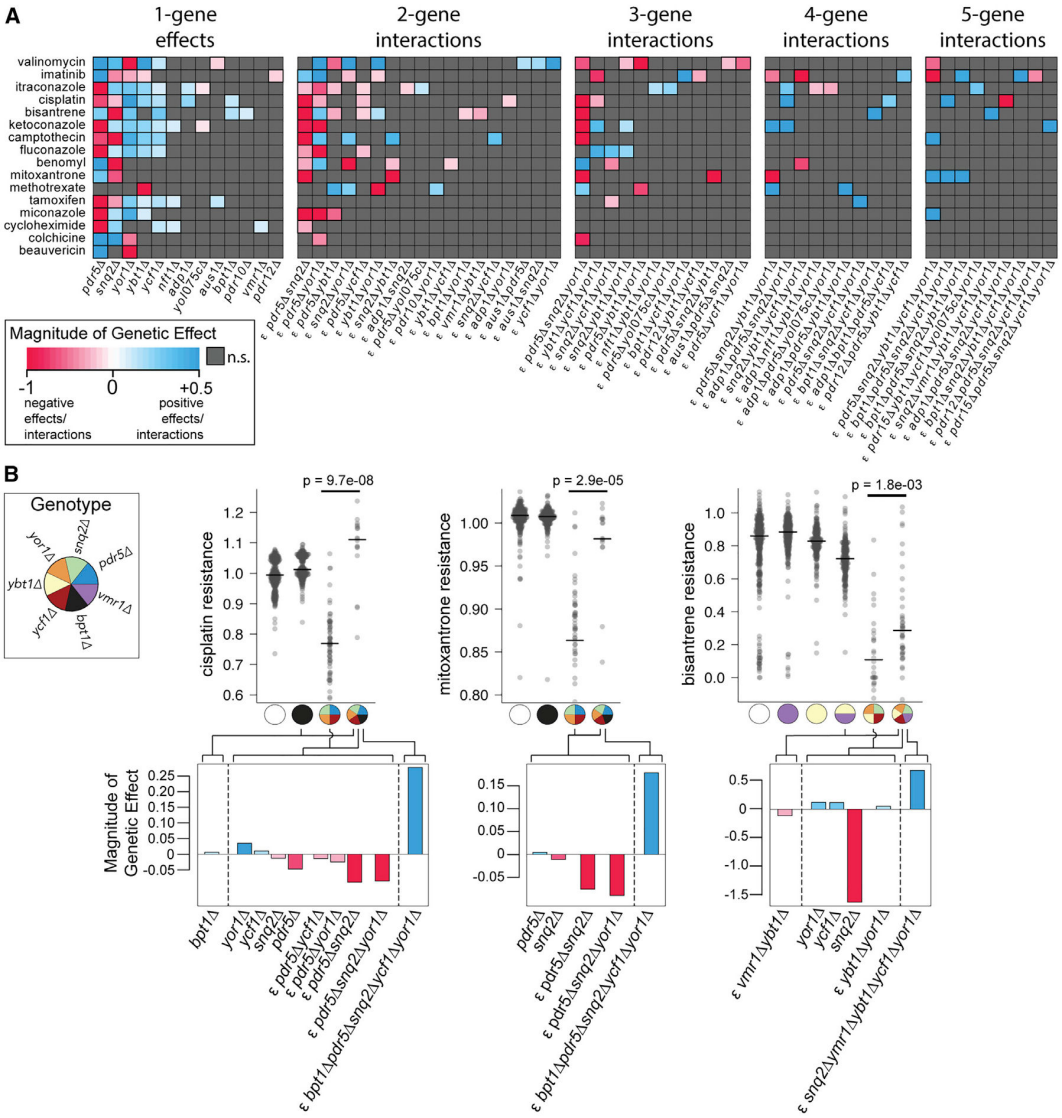
Drug-Dependent Complex Genetic Interactions among 16 ABC Transporters (A) All significant single-gene knockout effects and *X*-way genetic interactions (quantified by ε values) mediating resistance to each compound (Multiple-testing-adjusted p < 0.05). Magnitudes of genetic effects were estimated by a generalized linear model and then rescaled for each drug. (B) Illustration of three five-gene genetic interactions that were observed in cisplatin, mitoxantrone, and bisantrene experiments. For each illustrated interaction, strains were grouped by the five genes of interest (averaging over the 11 other loci). Top panels show distribution of drug resistance for strains in each group. Differences in median resistance (black lines) between the indicated four- and five-gene groups were evaluated via Mann-Whitney *U* test. The bottom row dissects the selected five-gene interactions by showing the magnitude of genetic effects for single-gene and lower-order knockout combinations.

To identify and model multi-gene knockout effects at all 16 transporters, we used a generalized linear model to capture both single-knockout effects and multi-gene interactions. To guard against overfitting for each drug, we considered only interactions between genes exhibiting marginal (single-gene) resistance or sensitivity to that drug and eliminated any terms that did not yield a statistically significant improvement in model fit (see STAR Methods). All single-knockout effects and multi-knockout genetic interactions that passed the significance test (p < 0.05 after adjusting for multiple testing) are shown in Figure 4A. The majority of these knockout effects and interactions (141/187, 75%) involved only the five frequently associated transporters (Figure 2A).

This analysis yielded genetic interactions involving two or more genes for 15 (94%) of 16 drugs examined (Figure 4A). The exception was beauvericin, for which we only recovered the previously reported sensitivity of *yor1Δ* knockouts (Shekhar-Guturja et al., 2016). Higher-order genetic interactions (involving three or more genes) were observed in 14 (88%) of 16 drugs tested (Figure 4A). Thus, XGA revealed higher-order genetic interactions for nearly all drug resistance phenotypes studied.

Of the 16 genes targeted by XGA, 14 were involved in at least one genetic interaction. Of these 14 genes, 13 were involved in at least one higher-order interaction. Remarkably, 11 of the 16 targeted genes were involved in at least one five-gene interaction.

Formally identifying complex genetic interactions captured many of the effects that had been readily apparent by manual examination of the five-gene fitness landscapes while yielding additional effects. For example, *pdr5Δ* was found to have a positive resistance effect under benomyl, a positive genetic interaction with *yor1Δ*, and a negative genetic interaction with *snq2Δ* (Figure 4A; Data S5). Strong high-order interactions involving genes outside of the five frequently associated transporters were also uncovered. In both cisplatin and mitoxantrone, for example, a five-way positive interaction pointed to the phenomenon that a *bpt1Δ* deletion confers resistance in a sensitive *pdr5Δ snq2Δ ycf1Δ yor1Δ* background (Figure 4B). Similarly, *vmr1Δ* conferred bisantrene resistance in a *snq2Δ ybt1Δ ycf1Δ yor1Δ* background while also conferring sensitivity in a *ybt1Δ* background (Figure 4B).

High-order genetic interaction analysis allowed finer parsing of the relationship between the involved genes. For example, the mitoxantrone sensitivity of the *snq2Δ pdr5Δ ybt1Δ yor1Δ* quadruple mutant (Figure 3) was modeled as the combination of two single-gene negative effects for *snq2Δ* and *pdr5Δ* alone, a two-gene negative interaction between *snq2Δ* and *pdr5Δ*, two three-gene negative interactions (between *snq2Δ pdr5Δ* and each of *ybt1Δ* and *yor1Δ*), and a four-gene {*snq2Δ*, *pdr5Δ*, *ybt1Δ*, *yor1Δ*} negative interaction (reflecting the fact that the quadruple mutant is more sensitive than would be expected given the observed resistance of any of the three-deletion subset genotypes; Figures 4A and 4B; Data S5). Together, these complex negative genetic interaction patterns suggest that the four genes enable mitoxantrone efflux in parallel. Similar “parallel efflux” genetic interaction patterns were observed, e.g., for {*pdr5Δ*, *snq2Δ*} in camptothecin and {*pdr5Δ*, *snq2Δ*, *yor1Δ*} in cisplatin (Figures 4A and 4B; Data S5).

### Using XGA to Learn Intuitive Genotype-to-Phenotype Models of the ABC Transporter System

While the generalized linear models used above do capture complex genotype-phenotype relationships, they do not always efficiently convey useful intuition about the system. For example, we reasoned that a set of transporter genes showing patterns of within-set negative genetic interactions suggests that each transporter is independently capable of drug efflux. Other genetic interaction patterns led us to conclude that the presence of one transporter can positively or negatively influence the activity of another (e.g., influence on Snq2 activity from *PDR5* and *YOR1* in benomyl). However, it is laborious to manually derive functional intuition from complex genetic evidence, and it is difficult to objectively evaluate the extent to which functional explanations fit the observed data. To demonstrate that complex genotype-phenotype relationships can be used to automatically and objectively derive biological intuition, we developed a neural network model.

**Figure 5.**
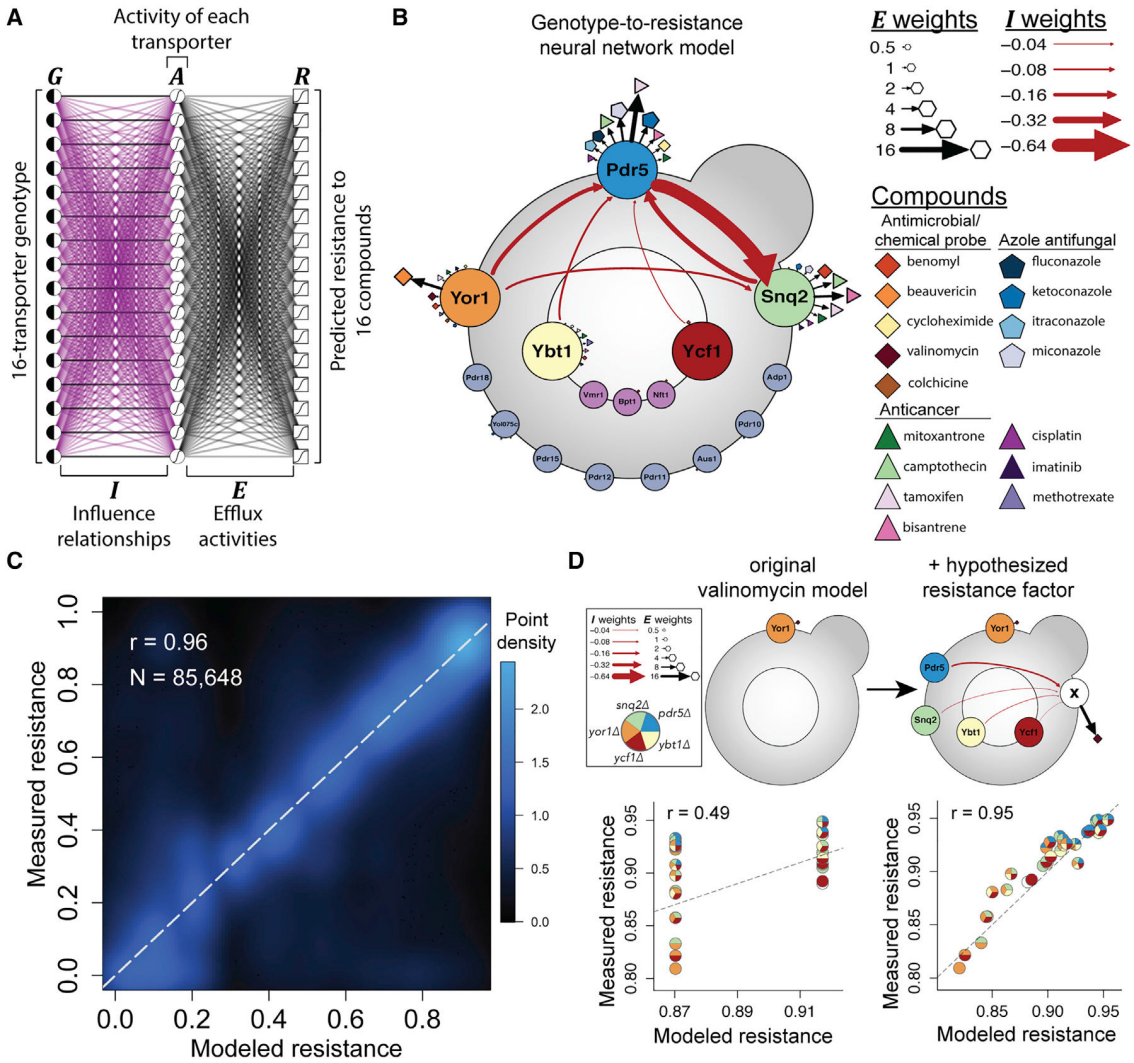
An Intuitive Neural Network Model of Complex Genotype-Phenotype Relationships (A) Structure of the neural network trained on XGA data. Genotypes (***G***) are provided as input. Transporter activities (***A***), transporter-transporter influence weights (***I***), and transporter-drug efflux weights (***E***) are inferred from the output drug resistance (***R***) values by back-propagation and stochastic gradient descent. (B) A schematic diagram of the weights learned by the neural network model after training. Inferred efflux activities (*E* weights) are represented as black arrows emerging from transporters. Each efflux arrow is labeled by a colored shape that indicates the effluxed drug. Inferred influences of one transporter on the activity of another (defined by ***I*** weights) are shown as intracellular arrows between transporters. All influences identified were negative. See also Figure S5A. (C) Agreement between measured and neural-network-modeled drug resistance. See also Figures S5B-S5D. (D) Extending the valinomycin resistance model improves agreement with measurement. For simplicity, data are shown only for the five “frequently associated” transporters. The trained neural network weights (top) are shown for the original model (top-left) and one with an extra node in the *A* layer to model potential influence on a hypothesized resistance factor (top right). See also Figure S5E.

We structured the neural network model (Figure 5A) to have three layers: (1) an input layer encoding the binary genotype for each of the 16 targeted transporters (***G***), (2) a middle “hidden” layer with values that estimate the activity of each of the 16 transporters (***A***; ranging from 0 to 1), and (3) an output layer that quantitatively describes resistance to each of 16 drugs (***R***; ranging from 0 to 1). To represent regulatory influence relationships between transporters, the links between genotype and activity layers have (initially unknown) “influence” weights (***I***), with positive weights where gene presence increases activity and negative weights where gene presence decreases activity. To enforce the expected behavior, that a transporter should not provide any efflux activity if it has been knocked out, and to associate each node in the activity layer with a specific gene, we explicitly set the activity of any knocked out transporters to 0. The links between activity and resistance layers have (initially unknown) non-negative “efflux” weights (***E***) that capture the extent to which each transporter can catalyze the efflux (or otherwise reduce the activity) of each drug. The model also allowed for offset terms for both ***A*** and ***R***.

We learned the appropriate network weights via back-propagation and stochastic gradient descent, training on the complete set of drug resistance phenotypes. To favor more parsimonious models and thus guard against overfitting, the cost function that was used to optimize network weights contained a penalty that acts to limit the number of non-zero weights, and each non-zero weight was tested for reproducibility and predictive impact (Figure S5A).

Training this model on our input dataset of 85,648 genotype-phenotype measurements yielded an interpretable neural network with only 71 non-zero fitted parameters (6 ***I*** weights, 49 ***E*** weights, no ***A*** offset terms, and 16 ***R*** offset terms). Despite its parsimonious nature, the trained neural network model largely recapitulated the observed genotype-phenotype relationships (*r* = 0.96, Figure 5C). To test that this performance generalizes to unseen data, we also trained the model using only data from one mating type then tested it using independent data from the other mating type. This yielded similar performance (*r* = 0.95 and *r* = 0.96 when using either mating type **a** or **α** as training, respectively [Figure S5B]), and the resulting independently trained models also showed strong agreement in parameter values (*r* = 0.98; Figure S5C), suggesting that model parameters were robustly determined.

The first notable result from this model was that all influence (***I***) values were either zero or negative. More specifically, only 6 out of 240 influence values were negative, while all others were zero (Figure 5B). Thus, while some ABC transporters exhibited negative influence on other ABC transporters, our study of 16 transporters in 16 drugs found no evidence that the presence of any ABC transporter can positively influence any other ABC transporter.

The three highest ***E*** weights were between Pdr5 and tamoxifen (***E*** = 13.2), Yor1 and beauvericin (***E*** = 8.6), and Snq2 and bisantrene (***E*** = 7.8). In each case, there was a strong sensitivity effect from removing these genes (Figure S4). However, the majority of the 49 non-zero ***E*** weights were of small effect (25/49 are below 0.5; Data S6).

The objectively trained neural network model provided functional intuition about complex genetic interactions that largely agreed with manual interpretations. For example, the manual genetic interpretation that Pdr5, Snq2, Yor1, and Ybt1 are each independently able to efflux mitoxantrone, was also supported by positive ***E*** links connecting each of these transporters to mitoxantrone (Figure 5B). The model showed Snq2 to have the highest mitoxantrone efflux activity (***E*** = 1.8), followed by Pdr5, Yor1, and Ybt1 (***E***= 1.5, 0.5, and 0.4, respectively; Figure 5B; Data S6). These differences were reflected in the fitness landscape: for example, while mitoxantrone resistance of a *ybt1Δ yor1Δ* deletion strain (Δ***E*** = −0.9) was not significantly different from the wild type (*p* = 0.12), deletion of genes encoding the two transporters with the highest inferred efflux (Snq2 and Pdr5, Δ***E*** = −3.3) yielded a 7% decrease in resistance (*p* = 1.2e-70). The ***I*** weights also pointed to differential inhibitory effects between transporters: for example, Snq2 activity is predicted to be more strongly inhibited by *PDR5* than by *YOR1* (***I*** = −0.69 versus −0.11, Figure 5B; Data S6), which is reflected, for example, by the observation that *pdr5Δ* yields greater benomyl resistance than does *yor1Δ* (Figure 3).

The neural network model also reflected the lack of genetic interactions observed for some drugs. For example, Yor1 was the only transporter modeled to provide strong efflux for beauvericin (***E*** = 8.6). We also did not find evidence for regulatory influence on Yor1 by the other transporters (Figure 5B; Data S6). This lack of influence and parallel efflux relationships mediating resistance to beauvericin was consistent with its lack of genetic interactions (Figure 4A).

While the neural network model was accurate overall, predictions departed systematically from observation for some drugs (Figure S5D). For example, while XGA showed that many multi-transporter deletions resulted in *increased* valinomycin resistance (Figure 3), the neural network only captured the decreased resistance resulting from *yor1Δ*, yielding poor predictions overall for the five-gene groups (*r* = 0.49, Figure 5D, left panel). Given previous reports of improved valinomycin resistance upon deletion of all 16 transporters (Suzuki et al., 2011) and effects on other genes upon multi-transporter deletion (Khakhina et al., 2015), we hypothesized that one or more of the transporters inhibits a valinomycin resistance factor outside of the 16 targeted genes.

To formally test whether inhibition of an unknown valinomycin resistance factor better captures the observed data, we extended the neural network model by adding a single hidden node to the ***A*** layer, allowing the neural network to model the hypothesized factor if the data support it. Training this extended neural network using valinomycin data substantially improved correspondence to the observed phenotypes (*r* = 0.95, Figure 5D, right panel) and yielded a model in which *SNQ2*, *PDR5*, *YBT1*, and *YCF1* each negatively influence an unknown valinomycin resistance factor. This model improvement was not simply the result of restricting the training procedure to valinomycin data but rather depended on the inclusion of this hypothesized factor (Figure S5E).

Taken together, examination of these neural network models provided intuition to explain 44/54 (81%) of the observed pairwise genetic interactions: 13 as arising from parallel efflux relationships, 26 as arising from influence relationships, and 5 as arising from effects on outside factors (Data S6).

### Deleting Four Genes Together Causes Synergistic *PDR5*-Dependent Fluconazole Resistance

**Figure 6.**
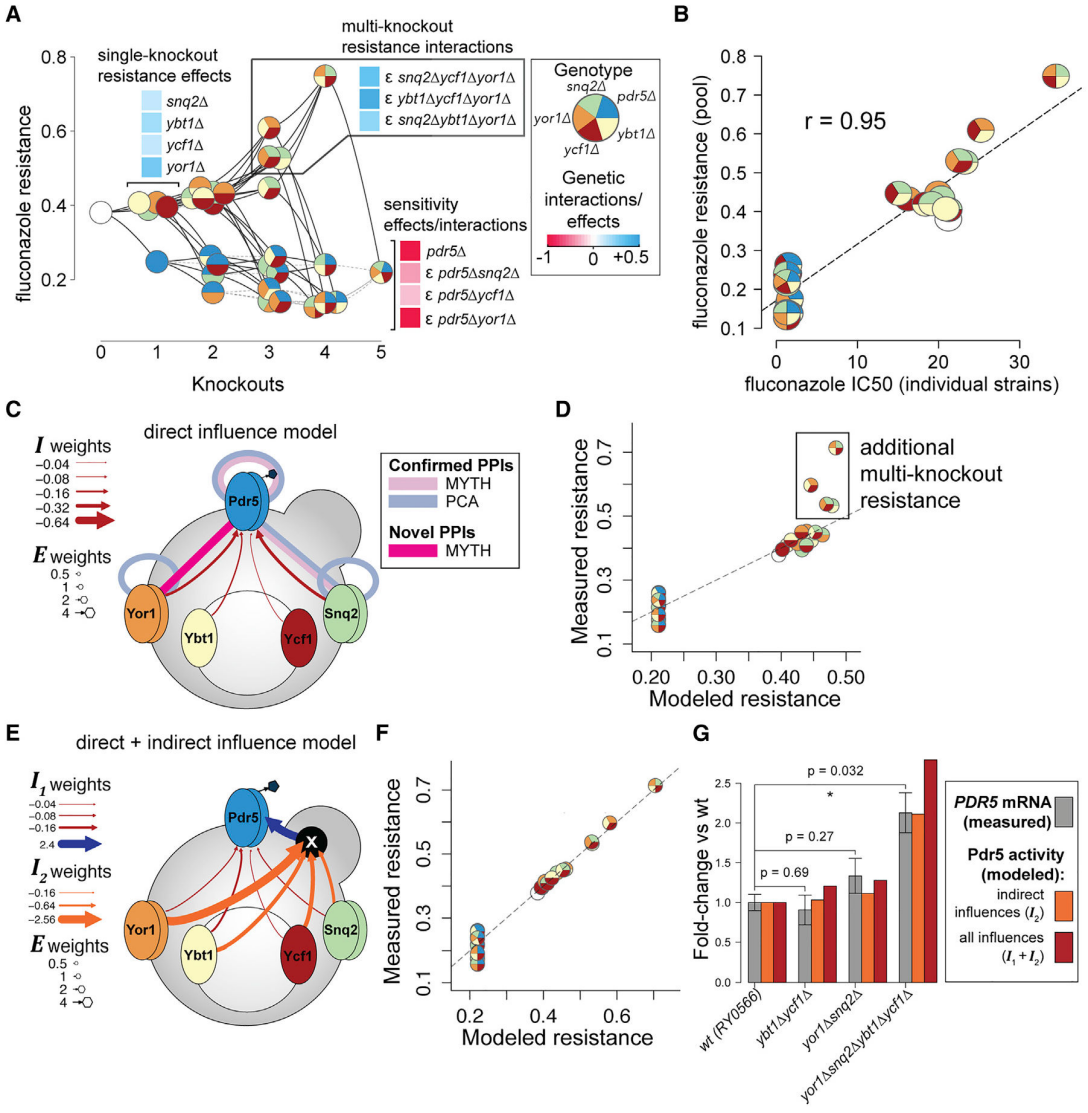
Deciphering a Complex Fluconazole Resistance Trait (A) Normalized fluconazole resistance is shown for strain groups corresponding to all combinations of five ABC transporter knockouts (as in Figure 3). Single-gene and interaction coefficients (from Figure 4) are highlighted for selected strain groups. (B) Measures of fluconazole resistance derived by *en masse* analysis of pooled strains agree closely with individually measured resistance (IC50) values in independently constructed strains. See also Figure S6A. (C) The neural network model (Figure 5B) predicts negative influence on Pdr5 by Snq2, Yor1, Ybt1, and Ycf1. Protein-protein interaction evidence supporting the plausibility of direct repression of Pdr5 by Snq2 and Yor1 is overlaid. This study confirmed all previously known PPIs shown, and revealed an unreported Pdr5-Yor1 PPI predicted by the direct interaction model. See also Figures S6B and S6C. (D) Highlighted multi-knockout strains show more resistance than predicted by the direct influence model. (E) An extended fluconazole resistance model captures both the direct (***I_1_*** weights) and indirect (***I_2_*** weights) influence of four transporters on Pdr5 activity. See also Figure S6D. (F) Modeling both direct and indirect influence improves prediction of resistant multi-knockout groups. (G) The synergistic effect on Pdr5 activity from deleting four ABC transporters is primarily explained by an indirect influence on *PDR5* transcript levels. Gray bars represent *PDR5* expression in the specified genotype, relative to that of wild-type, with error bars indicating standard error (n = 3). Significance was assessed by *t*-test. Colored bars show model-inferred Pdr5 activity (Figure 6E) for each genotype, relative to that of wild-type, considering all (red) or only the indirect (orange) influences.

One notable phenotype revealed by XGA was a quadruple deletion—*snq2Δ ybt1Δ ycf1Δ yor1Δ*—with high resistance to both fluconazole (Figure 6A) and ketoconazole (Figure S4). Further adding a *pdr5Δ* deletion to this quadruple mutant background restored fluconazole sensitivity to a level that was comparable with *pdr5Δ* alone. The quadruple-knockout resistance phenomenon was modeled as the combination of three positive three-gene interactions (all of the three-knockout combinations of {*snq2Δ*, *ybt1Δ*, *ycf1Δ*, *yor1Δ*} except *snq2Δ ybt1Δ ycf1Δ*), while its dependence on *PDR5* was modeled by three two-way negative interactions: {*pdr5Δ*, *snq2Δ*}, {*pdr5Δ*, *ycf1Δ*}, and {*pdr5Δ*, *yor1Δ*} (Figure 6A).

We confirmed these resistance observations in a more uniform genetic background by generating a single strain for each of the 32 possible combinations of *pdr5Δ*, *snq2Δ*, *ybt1Δ*, *ycf1Δ*, and *yor1Δ* knockouts (Figure S6A). The fluconazole resistance estimated from competitively grown XGA pools correlated well with measures obtained for individual strains—correlation was *r* = 0.95 with the fluconazole concentration expected to yield 50% inhibition (IC50; Figure 6B) and was *r* = 0.89 for fluconazole resistance (Figure S6A; STAR Methods). Consistent with pooled results, individual strain assays showed the *snq2Δ ybt1Δ ycf1Δ yor1Δ* strain to have the highest fluconazole resistance.

The neural network model indicated negative influence on Pdr5 from *SNQ2*, *YBT1*, *YCF1*, and *YOR1* (Figure 6C), thereby capturing the idea that *snqΔ ybt1Δ ycf1Δ yor1Δ* should be more resistant to fluconazole than strains carrying any subset of these knockouts. We wondered whether these negative influences stemmed from direct or indirect mechanisms. Given the known protein-protein interaction between Pdr5 and Snq2 (Snider et al., 2013) and previous reports of improved Pdr5-dependent drug resistance from knocking out *snq2Δ* or *yor1Δ* (Kolaczkowska et al., 2008), one might hypothesize that repression of *PDR5* from these two genes is mediated by direct interactions between transporters.

This hypothesis is further supported by previous reports that Pdr5 forms a homodimer (Snider et al., 2013; Tarassov et al., 2008), such that heterodimerization of Pdr5 and Snq2 transporters can draw subunits away from a homodimeric Pdr5 complex and thereby reduce Pdr5 efflux activity. Similarly, homodimers of Snq2 and Yor1 have also been reported (Snider et al., 2013; Tarassov et al., 2008). However, in addition to the known heterodimeric interaction between Pdr5 and Snq2, viewing our data through the lens of this model would also predict a previously unreported Pdr5-Yor1 heterodimeric interaction. Because all known protein interaction screening methods miss the majority of real interactions (Braun et al., 2009), we tested the predicted Pdr5-Yor1 interaction using two distinct assays: MYTH (Paumi et al., 2008; Snider et al., 2010) and PCA (Tarassov et al., 2008). Although PCA (Figure S6B) did not detect this Pdr5-Yor1 interaction, it was detected by MYTH (Figures 6C and S6C), thus confirming a key prediction of the direct repression model for the Pdr5-dependent decrease in fluconazole resistance provided by *YOR1*. All previously known MYTH and PCA interactions among Pdr5, Snq2, and Yor1 (including homodimers) were also recovered (Figures 6C and S6B and S6C).

While direct negative influence via protein interaction is an attractive model which successfully predicted an unreported protein interaction, previous studies suggest the importance of indirect negative influence at the transcriptional level. For example, a previous study found that while *pdr5Δ* and *yor1Δ* each resulted in increased benomyl resistance, combining them in a *pdr5Δ yor1Δ* strain resulted in non-additive *SNQ2* mRNA induction (Snider et al., 2013). Similarly, there is evidence for increased *PDR5* transcript levels in *yor1Δ snq2Δ* (Kolaczkowska et al., 2008). Such indirect multi-knockout transcriptional responses would result in non-additive influence effects that would not be well-captured by our original neural network. Indeed, while the neural network largely captured one- and two-knockout effects, there were several three- and four-deletion strains showing greater resistance than was captured in the model (Figure 6D).

To assess the potential importance of indirect negative influences, we extended the neural network model by adding a single hidden node between the ***G*** and ***A*** layers (Figure S6D). This extra node allows the neural network to capture more complex influence effects by learning (should the data support it) that these four genes can modulate the activity of a hidden “influence mediator”—e.g., the transcription factor complex Pdr1/3 known to regulate ABC transporters (Nawrocki et al., 2001)—which can in turn influence the activity of Pdr5. This extended neural network, trained using only fluconazole data, assigned substantial weights to the indirect influence mediator node (Figure 6E), and yielded fluconazole resistance that better modeled the unexpectedly resistant three- and four-knockout strains (Figure 6F). We confirmed that this improvement did not simply stem from training only on fluconazole data but rather required the indirect influence mediator node (Figures S6D and S6E).

To experimentally test whether the effect of the fluconazole-resistant quadruple mutant is explained (at least in part) by non-additive influence on *PDR5* transcript levels, we used qRT-PCR to measure *PDR5* mRNA levels in two double-knockout strains—*snq2Δ yor1Δ*, bearing deletions of two transporters localized in the plasma membrane, and *ybt1Δ ycf1Δ*, bearing deletions of two transporters localized in the vacuole—as well as the hyper-resistant quadruple knockout (*snq2Δ ybt1Δ ycf1Δ yor1Δ*). Based on the neural network model, *snq2Δ yor1Δ* and *ybt1Δ ycf1Δ* were expected to have only weak increases in Pdr5 activity relative to the wild-type (1.3× and 1.2×, respectively), while a strong increase (2.8×) was expected for *snq2Δ ybt1Δ ycf1Δ yor1Δ* (Figure 6G). Weaker effects were expected when considering only “indirect” influences from the hidden mediating factor (1.1×, 1.0×, and 2.1× for *snq2Δ yor1Δ*, *ybt1Δ ycf1Δ*, and *snq2Δ ybt1Δ ycf1Δ yor1Δ*, respectively) (Figure 6G). Using qRT-PCR, we found *PDR5* mRNA levels to be significantly higher in *snq2Δ ybt1Δ ycf1Δ yor1Δ* relative to the wild type (precisely the expected 2.1× increase; p = 0.032; Figure 6G) but not in either *snq2Δ yor1Δ* or *ybt1Δ ycf1Δ* (Figure 6G). Although a ~1.5× increase in *PDR5* mRNA levels had been previously reported for *snq2Δ yor1Δ* (Kolaczkowska et al., 2008), here the experimentally measured ~1.3× change did not achieve statistical significance (p = 0.27; Figure 6G), and the previous report of this phenomenon did not contain a statistical test. Overall, the observed *PDR5* expression changes were consistent with the relative indirect influence on activity expected from the extended neural network model.

Taken together, these results support the idea that both of two different influence mechanisms are occurring: one in which Snq2 and Yor1 each directly inhibit Pdr5 via protein interaction and another in which the presence of each of four transporter genes can indirectly inhibit Pdr5 activity via *PDR5* expression.

## DISCUSSION

Here we described XGA, a general strategy using systematic high-order combinatorial genetic engineering and multiplexed profiling to provide functional models of complex traits. Applying a specific genetic-cross-based implementation of XGA to 16 yeast ABC transporters uncovered complex genetic phenomena that were not evident from single and double gene knockout effects. Furthermore, XGA data enabled the generation of objectively-learned functional system models.

The XGA strategy, as we implemented it in yeast, can generate a combinatorially complex population by using individuals that differ at multiple loci. This cross-based approach contrasts SGA, which is efficient for generating double-mutant strains at many loci, but requires labor-intensive methods to individually engineer query strains with larger numbers of knockouts (Kuzmin et al., 2018). Similar cross-based methods have been applied extensively to pairs of outbred parents for mapping quantitative trait loci (QTLs) (Bloom et al., 2013). By using parents that differ only at a handful of positions, XGA can achieve much greater statistical power than QTL studies and can straightforwardly identify causal alleles by ensuring the absence of other proximal variants in genetic linkage. The use of engineered variants also allows XGA to study gene sets for which functional variation is not present in natural isolates (Lee et al., 2014).

As the genotyping strategy described here could also be applied to engineered point mutants, XGA could potentially be employed for a highly combinatorial study of prioritized QTL variants (Sadhu et al., 2016) in a more uniform genetic background. As natural variants can lead to gain-, change-, or reduction-of-function as opposed to strictly loss-of-function mechanisms for the null alleles that we studied, modeling natural variation will require learning the activity and influence of specific alleles from data, as opposed to setting these to zero as we did for knockout alleles. XGA could also be used to evaluate the evolutionary accessibility of alternative “trajectories” of genotypic change (e.g., Figures 2D, 2E, and 3) that transition from one combinatorial genotype to another via serial addition of successive naturally occurring mutations (Ferretti et al., 2018).

A cross-based XGA strategy has broader potential for use with multiple variants of other gene sets. For example, there are 80 yeast gene families in *S. cerevisiae* with six or more genes that might be studied using multiple deletions (Suzuki et al., 2011). Other functionally related sets of genes can also be studied with XGA, as many such gene sets are likely to be strongly enriched for both pairwise and three-gene genetic interactions (Costanzo et al., 2016; Kuzmin et al., 2018). For example, XGA could be performed using an existing yeast mutant with 16 pheromone-response pathway genes deleted (Shaw et al., 2019). Recent advances in methods for more routine construction of multi-mutant strains lend themselves to future applications of XGA. For example, CRISPR has been used to introduce variation into yeast cells at up to five loci with a single transformation (Jakočiūnas et al., 2015). Simultaneous variant engineering at 3–6 loci has been described in multicellular organisms, e.g., in mouse (Wang et al., 2013), zebrafish (Jao et al., 2013), *C. elegans* (Xu et al., 2016), and *Arabidopsis* (Zhang et al., 2016). As it may not be convenient (or compatible with viability) to introduce all targeted variants within a single individual, targeted variation may instead be distributed between two parental strains (or more with the use of multi-generational crosses).

Future implementations of XGA might involve directly engineering a population of cells with diverse combinatorial changes (Wong et al., 2016; Zeitoun et al., 2017), without the need for genetic crosses. For example, pooled approaches to profile two-gene combinatorial mutants in human cells (Horlbeck et al., 2018; Najm et al., 2018; Shen et al., 2017; Wong et al., 2016) may be expanded to allow for higher combinatorial complexity. In *E. coli* and yeast, methods have been designed to combinatorially modify multiple loci in a population of cells (DiCarlo et al., 2013; Wang et al., 2009), and these may be extended to allow large-scale strain isolation and phenotyping (Zeitoun et al., 2017, 2015). Single-cell barcoding methods (Dixit et al., 2016) may also permit sampling a greater number of genotypes—here, we sampled ~8% of 65,536 knockout combinations at 16 genes, while single-cell barcoding methods might be adapted to profile ~10^5^ haploid strains, achieving similar depth for 20 genes.

Many future expansions can be envisioned for the application of XGA to yeast ABC transporters. Here we showed that a small set of bioactive small molecules, many of which were selected without prior knowledge of transporter-substrate relationships, revealed many roles for *PDR5*, *SNQ2*, *YOR1*, *YBT1*, and *YCF1*. The population engineered in this study readily allows XGA of these 16 yeast ABC transporters using additional compounds, such that using known transporter-substrate affinities may better reveal roles for the remaining genes. Additional genes could also be deleted in each pool *en masse*, thereby expanding XGA targets (e.g., to include *PDR1* and *PDR3*, which transcriptionally control several ABC transporters). Higher-content phenotyping approaches could also provide a richer profile of the cellular response to ABC transporter perturbation (Khakhina et al., 2015).

In our application of XGA to yeast ABC transporters, all influences were negative. There is also evidence for negative influence between ABC transporters in mammals. For example, *ABCC3* increases in expression when *ABCC2* is disrupted in Dubin-Johnson Syndrome (Donner and Keppler, 2001; König et al., 1999), and *ABCG5* and *ABCG8* both increase in expression when *ABCG2* (a gene that confers breast cancer xenobiotic resistance in humans) is knocked out in mice (Huls et al., 2008). However, there is also evidence that mammalian ABC transporters can positively influence each other. For example, ABCA12 improves the stability and abundance of ABCA1 (Fu et al., 2013). In another example, ABCG5 and ABCG8 form a functional heterodimer, such that each transporter requires the other for activity (Graf et al., 2003). Thus, an analogous XGA of human ABC transporters could yield better understanding of the involvement and interplay of these genes in metabolite or catabolite transport and drug resistance.

Here, we used a neural network to infer function from phenotypic profiles of combinatorial genotypes. Complex genotype-phenotype data have often been viewed as a network, with edges either representing genetic interactions or similarity between genetic interaction profiles (Costanzo et al., 2010). While these representations have been useful, new approaches are needed to convey functional information from the phenotypes of higher-order mutant combinations. It seemed clear that among four different ways to visualize XGA results (Figures 2D, 3, 4, and 5B), here the “visible neural network” model inspired by previous work (Ma et al., 2018) was the most useful and intuitive representation. Where epistasis analysis has been more narrowly focused on determining the order of genes within pathways (Angeles-Albores et al., 2018; Boettcher et al., 2018; St Onge et al., 2007), here we could quantitatively model relationships between ABC transporters that did not clearly follow an ordered pathway.

The neural network structure we employed may be appropriate for other transport processes, but future XGA studies will generally need to tailor neural networks that model genetic interactions based on prior understanding of gene functions, including a consideration of potential unobserved factors. For example, it has been shown that two mutations in a given protein can additively affect thermodynamic stability to yield non-additive effects on overall function (Diss and Lehner, 2018; Sarkisyan et al., 2016). Here, we modeled an analogous phenomenon, that independent effects of transporter knockouts on two unobserved factors—drug efflux activity and between-transporter influence—can have non-linear effects on phenotype, resulting in complex genetic interactions. In the case of fluconazole resistance, a set of complex genetic interactions involving five transporters could be simply modeled as one fluconazole-effluxing transporter (Pdr5) and four transporters that each independently modulate an unobserved Pdr5 regulator (Figure 6E).

In summary, we described a general XGA strategy for profiling and modeling high-order genotype-to-phenotype relationships, implemented a version of XGA in yeast, and showed that it can help functionally dissect and understand a complex system.

## STAR★METHODS

### LEAD CONTACT AND MATERIALS AVAILABILITY

Further information and requests for resources and reagents should be directed to and will be fulfilled by the Lead Contact, Frederick P. Roth (fritz.roth@utoronto.ca). All unique/stable reagents generated in this study are available from the Lead Contact without restriction.

### EXPERIMENTAL MODEL AND SUBJECT DETAILS

#### Saccharomyces Cerevisiae Strains

##### RY0622 (ABC-16/’Green Monster’ MATa)

MAT**a** adp1Δ snq2Δ ycf1Δ pdr15Δ yor1Δ vmr1Δ pdr11Δ nft1Δ bpt1Δ ybt1Δ pdr18Δ yol075cΔ aus1Δ pdr5Δ pdr10Δ pdr12Δ can1Δ::GMToolkit-**a** (CMVpr-rtTA KanMX4 STE2pr-Sp-his5) his3Δ1 leu2Δ0 ura3Δ0 met15Δ0

##### RY0146 (“Toolkit-a” Strain)

MAT**a** lyp1Δ his3Δ1 leu2Δ0 ura3Δ0 met15Δ0 can1Δ::GMToolkit-a (CMVpr-rtTA KanMX4 STE2pr-Sp-his5)

##### RY0566 (“Toolkit-a” Strain with Tet-inducible GFP-URA3)

MAT**a** lyp1Δ his3Δ1 leu2Δ0 ura3Δ0 met15Δ0 can1Δ::GMToolkit-aA (CMVpr-rtTA KanMX4 STE2pr-Sp-his5) hoΔ::tetO2-GFP-URA3

##### RY0148 (“Toolkit-α” Strain)

MAT**α** lyp1Δ his3Δ1 leu2Δ0 ura3Δ0 met15Δ0 can1Δ::GMToolkit-α (CMVpr-rtTA NatMX4 STE3pr-LEU2)

##### Barcoded RY0148 Pool

MAT**α** lyp1Δ his3Δ1 leu2Δ0 ura3Δ0 met15Δ0 can1Δ::GMToolkit-α (CMVpr-rtTA NatMX4 STE3pr-LEU2) hoΔ::loxP UP-tag HphMX4 DN-tag lox2272

### METHODS DETAILS

#### Creating the Barcoder Plasmid

We added a ‘barcoder’ locus flanked by *loxP* and *lox2272* into a pSH47 plasmid backbone expressing GAL1pr-CRE. This barcoder locus consisted of a random 25bp DNA sequence (‘UP tag’) in between two common primer regions (‘US1’ and ‘US2’), followed by a HphMX4 cassette, and another random 25bp DNA sequence (‘DN tag’) in between two common primer regions (‘DS1’ and ‘DS2’). This entire locus was flanked by *loxP* and *lox2272* sites.

To construct this locus, a barcoded HphMX4 construct was first created (Figure S1A). HphMX4 was amplified from a pIS420 plasmid using the STEP1F and STEP1R primers containing HphMX4 homology and US2/DS1 overhangs (Data S1). The PCR program used for this step was 98°C for 30sec; 25 cycles of 98°C for 10sec, 59°C for 10sec, 72°C for 60sec; 72°C for 5min; hold at 4°C. These PCR products were purified using a QIAprep Spin Miniprep Kit (QIAGEN, 27106) and confirmed using 2% gel electrophoresis. To add the random barcodes and US1/DS2 regions to the resulting HphMX4 amplicon, the STEP2F and STEP2R primers were used with the following PCR program: 98°C for 30sec; 25 cycles of 98°C for 10sec, 68°C for 10sec, 72°C for 60sec; 72°C for 5min; hold at 4°C. These resulting products were again purified using a QIAprep Spin Miniprep Kit and ~1.5-1.6kb products were confirmed using 2% gel electrophoresis. To add *loxP/lox2272* sites, PCR was performed with the STEP2 products using the SacI-loxP-HphMX4-Barcode-F / SacI-lox2272-HphMX4-Barcode-R primers. The PCR program used for this step was: 98°C for 30 sec; 26 cycles of 98°C for 15sec, 64°C for 20sec, 72°C for 65sec; 72°C for 5min; hold at 4°C. The resulting PCR products were purified using a QIAprep Spin Miniprep Kit, and ~1950bp products were confirmed using 2% gel electrophoresis.

To confirm correct synthesis of the barcoded HphMX4 construct, two PCR reactions were performed on the resulting products. The first PCR reaction was performed with the SacI Reamp F/US2 primer pairs, and the second was performed using DS1/SacI Reamp R primer pairs. The PCR program used for both of these reactions was: 98°C for 30sec; 25 cycles of 98°C for 10sec, 59°C for 15sec, 72°C for 30sec; 72°C for 5min; hold at 4°C. Expected sizes (~132bp, 137bp) were confirmed using 4% gel electrophoresis. All of the above PCR reactions were performed using High Fidelity Phusion Master Mix (NEB, M0531).

To prepare for cloning of the barcoder locus, pSH47 was digested with SacI using 100μl of 250ng/μl pSH47, 100μl NEBuffer 4 (NEB, B7004S), 10μl BSA (NEB, B9000), 10μl SacI-HF in 1ml sterile water. 100μl of this mixture was incubated at 37°C for two hours, and inactivated by incubation at 65°C for 20min. Digest products were purified using a QIAprep Spin Miniprep Kit and confirmed using 0.8% gel electrophoresis.

#### Generating a Barcoder Strain

A linear *URA3* cassette flanked by *loxP* and *lox2272* sites and homology to the *HO* gene was amplified from purified pIS418 with the 5’HO-loxP-URA and URA-lox2272-3’HO primers using the following PCR program: 98°C for 30sec; 25 cycles of 98°C for 10sec, 60°C for 10sec, 72°C for 70sec; 72°C for 5min; hold at 4°C. This PCR reaction was performed using High Fidelity Phusion Master Mix and was purified using a QIAprep Spin Miniprep Kit. This cassette was integrated into the *HO* locus of the RY0148 strain through transformation using an EZ transformation kit (Zymo Research, T2001), to serve as the ‘landing pad’ for barcode integration. Transformants selected for growth in SC –Ura plates, and were later verified to exhibit no growth in 5-FOA. A transformant was selected to confirm *HO* locus integration using three PCR reactions with the following primer pairs: 5’HO-URAreamp + midURA-5’; 5’HO-URAreamp + midURA-3’; 5’HO-URAreamp + 3’HO-URAreamp. All PCR reactions were performed using High Fidelity Phusion Master Mix with the following program: 98°C for 30sec; 25 cycles of 98°C for 10sec, 50°C for 10sec, 72°C for 70sec; 72°C for 5min; hold at 4°C. Expected PCR product size was confirmed using 2% gel electrophoresis.

The *HO*::*loxP-URA3-lox2272* integrant strain was then transformed with a mixture of digested pSH47 and purified PCR products (Figure S1B) to enable in-yeast-assembly (Gibson et al., 2009). Transformation was carried out using a previously established protocol (Gietz and Schiestl, 2007), with a ~1:6 mixture of digested pSH47:HphMX4 barcode cassette (~12μg digested pSH47 and 15μg cassette). Transformants were grown at 30°C in YPG +HygroB plates for 3 days, allowing both selection of successful transformants and Gal1p-Cre induction. These cells were then scraped and grown overnight in 5-FOA plates to select against non-recombinant strains and strains containing the barcoder plasmids.

Twenty colonies were confirmed to have barcode integration using PCR and Sanger sequencing. Lysates were made by mixing a sample of each colony with 2μl Sterile DNA Free Water, 2μl 0.2M pH 7.4 Sodium Phosphate Buffer, 0.5 μl 5U/μl Zymo Research zymolyase and incubated at 37°C for 25min and 95°C for 10 min, and stopped by adding 125μl of sterile DNA-free water. To each lysed colony, two sets of primer pairs to verify the strain barcode-specific UP and DN tag - US2 and a sequence complementary to 5’ of the *HO* gene (5’HO); DS1 and a sequence complementary to the 3’ of the *HO* gene (3’HO), using the following program: 98°C for 30sec; 25 cycles of 98°C for 10sec, 59°C for 15sec, 72°C for 30sec; 72°C for 5min; hold at 4°C. PCR reactions were performed using High Fidelity Phusion Master Mix and analyzed using gel electrophoresis. EXOSAP-IT purification (Thermo Fisher, 78201) was performed on the PCR products, and they were Sanger sequenced with the 5’HO seq and 3’HO seq primers to confirm the correct barcode construct.

#### Creating a ‘Gold Standard’ Genotyped Set

To create a ‘Gold Standard’ genotyped set, 40 progeny strains (19 MAT**a** and 21 MAT**α**) were subject to individual strain genotyping. For these 40 strains, and for an RY0148 isolate, the strain-specific UP and DN tags were also PCR-amplified using two sets of primers and subjected to Sanger sequencing as above.

To genotype each strain at the 16 ABC transporter loci, two PCR reactions were performed for each locus - one to determine the presence of a GFP integration cassette, and another to determine the presence of the wild type gene, as previously described (Suzuki et al., 2011). For the cassette confirmation reactions, locus–specific PCR primers from the 5′ flanking sequences of each gene were paired with a common primer complementary to the *GFP* cassette (Data S1). Gene presence confirmation primers were designed individually for each gene (Data S1). PCR reactions were performed with a Platinum PCR SuperMix High Fidelity (Thermo Fisher, 12532016) using the following program: 94°C for 2min; 34 cycles of 94°C for 30sec, 55°C for 30sec, 68°C for 60sec; 68°C for 10min; hold at 4°C. PCR products were analyzed using gel electrophoresis.

#### Generating Barcoded Random Knockout Progeny

Mating, sporulation, and haploid selection was performed between the RY0622 ‘Green Monster’ strain (MAT**a**) and the barcoded RY0148 pool (MAT**α**) as previously described (Suzuki et al., 2011), selecting for MAT**a** and MAT**α** progeny separately. The two pools were then grown in YPD +HygroB to select for barcoded haploids. The SC–Leu pool was further grown in SC–Ura to select against barcoder strain parents that may have escaped diploid selection. Using a QPix™ 400 Microbial Colony Picker (Molecular Devices), 5,461 MAT**a** and 5,461 MAT**α** colonies were picked onto 384 well plates. In addition, 299 known positions in both the MAT**a** and MAT**α** arrayed collections consisted of known strains – either one of 40 ‘Gold Standard’ genotyped strains, RY0148, or RY0622 – to act as genotyping controls (Data S2).

#### Pooled Strain Genotyping

A previously-described Row-Column-Plate (RCP)-PCR protocol (Yachie et al., 2016) was adapted in order to perform *en-masse* genotyping of the random knockout progeny using high throughput sequencing. This protocol first uniquely tags PCR products originating from the same well on a given plate, by the use of a 5’ tag encoding the well row (R) in forward primers, and a 3’ tag encoding the well column (C) in the reverse primers (Yachie et al., 2016). Additionally, these primers contain a linker sequence (PS1 or PS2) which were used subsequently to amplify barcode locus amplicons that have been pooled for each plate while incorporating indices that encode the plate of origin (Data S1).

For each well in the collection, lysates were made on a new set of plates. 4 μl of overnight yeast culture was mixed with 8 μL 0.2 M sodium phosphate buffer (pH 7.4), 4 μl DNA free dH2O,0.05 μl 5 U/μl zymolyase (Zymo Research, E1005) and incubated at 37 °C for 35 minutes. 64 μl DNA free dH2O was added to each well to prepare PCR template.

Four ‘Row-Column’ PCR reactions were performed on the lysates with the following primer pairs: PS1+R+U1 and PS2+C+U2 to amplify DNA barcodes encoding the UP tags for each gene deletion; PS1+R+D1 and PS2+C+D2 to amplify the deletion-specific DN tags; PS1+R+US1 and PS2+C+US2 to amplify the strain-specific UP tag; PS1+R+DS1 and PS2+C+DS2 to amplify the strain-specific DN tag (Data S1). PCR reactions were performed with 2 μl of lysed colonies using a Hydrocycler Thermal Cycler (KBioscience) with the following program: 95 °C for 5 min; 23 cycles of 95 °C for 60 sec, 57 °C for 35 sec, 72 °C for 45 sec; 72 °C for 2 min; hold at 4 °C. Row-Column PCR products from each plate were pooled and size was verified on a 4% agarose gel. PCR products from each plate were combined, and Illumina adapters containing plate-identifying tags were added using an additional PCR reaction as previously described (Yachie et al., 2016). A pair of PXX_PE1.0 and PYY_PE2.0 primers (Data S1) were added to 3-6 μl pooled products (calibrated to ~150 ng) from each plate to encode the plate of origin, and were amplified using the following PCR program: 98 °C for 30 sec; 15 cycles of 98 °C for 10 sec, 59 °C for 15 sec, 72 °C for 40 sec; 72 °C for 2 min; hold at 4 °C. All PCR reactions above were performed using High Fidelity Phusion Master Mix.

Expected product size from the plate tags was confirmed on 4% agarose gel. PCR products were purified using a Qiagen MinElute Gel Extraction kit (QIAGEN, 28604), and qPCR was performed on all plate tag PCR products using a LightCycler 480 (Roche) and KAPA SYBR FAST qPCR Kit (Roche). qPCR results were used to generate a pool with approximately equal amounts of each sample, and 100 μl of this multiplexed sample were run on a 4% gel. Products of the desired size (260-290 bp) were isolated from each lane, purified using a QIAGEN MinElute Gel Extraction kit, and another qPCR was run on the purified sample.

#### Analysis of Pooled Strain Genotyping Data

Pooled strain-genotyping PCR products were sequenced using an Illumina HiSeq 2000, and the reads were demultiplexed into individual samples corresponding to a plate and well of origin.

For each sample, a genotype calling pipeline determined the strain-specific tag sequences and genotype from the reads. The parameters of this pipeline were trained based on known reference strains. Cross-validated accuracy for each gene is reported in Figure S1C.

UP or DN tag identity at the strain-identifying barcode locus and a corresponding genotype was successfully determined for 7,195 samples. For 7,030 samples, either the UP or DN tag was unique, while for 165 samples, both of the strain-identifying UP and DN tag sequences were the same as those in another sample where the called genotype was isogenic or highly similar (≤ 2 differences), indicating the presence of a single strain in multiple wells. Where genotypes were highly similar, one of the genotypes was randomly assigned to the strain-identifying-barcode sequences.

#### Genotype Refinement

For 131 MAT**α** and 73 MAT**a** strains, pooled sequencing analysis had called the genotype as wild-type. Many of these strains were isolated and tested for the presence of one or more gene knockout cassettes by growth in SC–Ura. Of 96 MAT**α** strains tested, 74 exhibited no detectable growth in SC–Ura (indicating the absence of any knockout cassettes), and likely arose from remaining barcoder parents which had escaped a previous SC–Ura selection step (Data S2). The genotypes for these 74 strains were kept as is, while the other 23 strains, as well as 46 untested strains were discarded from the analysis (Data S2). Out of 45 MAT**a** strains, all exhibited growth in SC–Ura (indicating a knockout cassette at one or more loci, Data S2). Individual genotyping was performed for these MAT**a** strains, and was successful for 40 of 45 strains, confirming the lack of true wild types. These strains had their genotype corrected (Data S2). The 5 unsuccessfully genotyped strains, as well as 28 apparently-wild-type but untested MAT**a** strains were discarded from analysis. When calculating linkage and distribution of gene knockouts (Figure S1D), the wild-type MAT**α** strains were also excluded from analysis.

#### Secondary Estimate of Genotyping Accuracy

To lend independent support to the genotyping accuracy determined by gold standard strains, an alternate method based on the distribution of knockouts in the population was used. Since *en masse* genotyping associates barcode sequences with ABC transporter knockouts, the absence of a given barcode implies either a wild-type genotype at that locus or a failure in amplification, sequencing, or calling. Conversely, cases where a wild-type is assigned a mutant genotype are expected to be comparably rare. Excess wild-type calls lead to a reduction in the average number of knockouts in the pool, and can be used to estimate genotyping accuracy. The average number of knockouts in the pool was 7.0, lower than the 8 expected with perfect genotyping. If wild-type to mutant miscalls are negligible, this number is most likely with an ‘asymmetric’ genotyping accuracy of 93.8%, compared to the 93.2% estimated by comparison to gold standards (Figure S1D).

#### Individual Liquid Growth Profiling

To measure individual strain growth, 100μl of starting culture at 0.0625 OD600_nm_ was grown in a 96 well-plate in a temperature-controlled shaking spectrophotometer (Tecan GENios microplate reader). Growing cultures were shaken at 800 rpm at 30°C and OD600_nm_ of each well was measured every 15 min.

#### Pool Growth Profiling by Barcode Sequencing

Progeny with at least one mapped strain-identifying barcode (Data S2) were combined into two separate liquid YPD + 15% glycerol pools separated by mating type, and kept at −80°C. Samples from the original YPD + glycerol pool were thawed and added to the appropriate drug or DMSO solvent-containing medium at a final concentration of 0.0625 OD_600 nm_ in 10ml. In addition, a ‘0 generation’ sample was immediately harvested from the YPD + glycerol pool and processed for DNA extraction and sequencing. After growth to approximately 2 OD_600 nm_ (~5 generations), cells were collected and processed for sequencing, and a small aliquot was diluted in fresh media (at a final concentration of 0.0625 OD_600 nm_ in 10ml) in presence of drug or solvent to be grown for an additional 5 generations. This process was performed twice – once with sequenced samples corresponding to approximately 0, 5, and 15 generations, and a second time with sequenced samples corresponding to approximately 0, 10, and 20 generations.

Harvested samples were subjected to genomic DNA extraction using a YeaStar™ Genomic DNA Kit (D2002, Zymo Research), quantified using the Quant-IT dsDNA BR Assay kit (Invitrogen, Q32853), and diluted to a final concentration of 25 ng/μl. 350ng of DNA from each sample was indexed with the following PCR mixture: 20 μl of 2x Platinum PCR SuperMix High Fidelity, 1 μL of 10 μM F primer, and 1 μl of 10 μM R primer. F and R primer pairs were PXX+US1/PYY+US2 and PXX+DS1/PYY+DS2 for the strain-specific UP and DN tag, respectively. PXX and PYY correspond to sequences containing plate-specific Illumina sequencing adapters, as well as tags which were used to demultiplex the samples (Data S1). PCR products were amplified using the following program: 98 °C for 30 sec; 24 cycles of 98 °C for 10 sec, 60 °C for 10 sec, 72 °C for 1 min; 72 °C for 5 min; hold at 4 °C. After indexing, equal volumes of UP-tag and DN-tag PCR products from each pool were run on a 3% agarose gel. The expected 210bp bands were isolated and purified using a QIAGEN MinElute Gel Extraction kit. DNA size and purity were confirmed by Agilent Bioanalyzer High Sensitivity DNA kit (5067-4626). DNA yield was quantified in triplicate using a KAPA SYBR FAST Universal qPCR kit (KK4824). Approximately equal amounts of each sample were combined and sequenced using an Illumina NextSeq 500 High Output v2 kit.

#### Targeted Mating to Obtain 32 Knockouts

The TWAS21230902 strain (genotyped as *pdr10Δ pdr18Δ pdr5Δ snq2Δ ybt1Δ ycf1Δ yor1Δ* by RCP-PCR; Data S2) was subject to individual strain genotyping (Suzuki et al., 2011), which confirmed the expected wild-type and knockout PCR products at each locus. This strain (MAT**α**) was mated with RY0566 (MAT**a**), and was subject to sporulation and MAT**a** haploid selection (Suzuki et al., 2011). Individuals from this cross were arrayed onto 96 well plates, and individually genotyped at *PDR10* and *PDR18*. Strains with no deletions at these genes were further genotyped at *PDR5, SNQ2, YBT1, YCF1*, and *YOR1*. PCR reactions for individual genotyping of these progeny used the QIAGEN Multiplex PCR Plus Kit (206152) with the following program: 95°C for 5min; 34 cycles of 95°C for 30sec, 57°C for 30sec, 72°C for 30sec; 68°C for 10min; hold at 4°C. After analysis of genotyping results, one strain of each genotype combination was chosen to create the 32-strain collection. These chosen 32 strains were again individually genotyped at these 5 loci for validation.

#### Pre-processing Data from BarSeq Assays

Paired-end Illumina sequencing data were first de-multiplexed by searching for an exact match to the tag regions of the PXX and PYY primers within each pair of reads. For each read in each de-multiplexed sample (corresponding to a combination of mating type, timepoint, and drug), strain identification is attempted by searching a reference database of all barcodes matching the sample mating type. If an exact match is not found, up to two ungapped mismatches are permitted to assign a putative strain identity, which is then accepted if there are at least 2 additional mismatches separating this identity with the next closest match (e.g. if 2 mismatches are present with the closest match, then the next closest match must have 4 or more mismatches). This process was performed for both the forward and reverse reads (corresponding to the UP and DN tags) for each strain (potential cases where the putative strain identity differed between tags were discarded). All samples for which fewer than 200,000 reads could be mapped either to an UP or DN tag were discarded. Because t = 0 samples were collected twice, counts from both runs were summed.

#### Deriving Resistance Measures from BarSeq

After pre-processing BarSeq data, a count *c*_*t*,*s_x_*,*d*_ was derived at each timepoint *t*, for each strain *s*_*x*_, in each drug pool *d*. Each count in each sample was then converted to a frequency *f*_*t*,*s_x_*,*d*_ with division by the total count across all strains in that sample:

$$f_{t,s_{x},d}=\frac{c_{t,s_{x},d}}{\sum_{i=1}^{n}c_{t,s_{i},d}}$$

If both an UP and DN tag for a given strain were successfully linked to a genotype, *f*_*t*,*s_x_*,*d*_ estimates were calculated separately with the counts from each tag, and the resulting *f*_*t*,*s_x_*,*d*_ estimates were averaged. Otherwise, only the available tag was used for this calculation.

Using these counts, we aimed to estimate an exponential growth rate for each strain under each drug $\left({\hat{g}}_{s_{x},d}\right)$. Here, ${\hat{g}}_{s_{x},d}$ represents the expected number of doublings per given time-point. First, we model the expected abundance of each strain in a drug at a time point $\left({\hat{A}}_{t,s_{x},d}\right)$, given an exponential growth rate and initial abundance (***A***_0,*s_x_*,*d*_).

$${\hat{A}}_{t,s_{x},d}=A_{0,s_{x},d}2^{{\hat{g}}_{s_{x},d}t}$$

Using barcode counts, we measured *f*_0,*s_x_*,*d*_. This frequency is proportional to an absolute starting abundance metric (***A***) for each strain (e.g., number of cells). Therefore, the above relationship can be restated as:

$${\hat{A}}_{t,s_{x},d}=(k\cdot f_{0,s_{x},d})2^{{\hat{g}}_{s_{x},d}t}$$

We use this relationship to fit ${\hat{g}}_{s_{x},d}$ to the observed abundance data (***A***_*t*,*s_x_*,*d*_). To calculate abundance, we use frequency at each time point, multiplied by the expected relative cell count of the pool compared to time 0. We define *t* as the number of pool generations since *t* = 0, so that the relative abundance vs *t* = 0 can be expressed as 2^*t*^. For example, a strain with with 1/100 frequency will correspond to N/100 cells (where N is the starting number of cells) at t = 0, but 2N/100 cells after one generation of growth, since the total number of cells in the pool have doubled once. The same rescaling constant *k* can be used to obtain the same units as $\hat{A}$:

$$A_{t,s_{x},d}=(k\cdot f_{t,s_{x},d})2^{t}$$

To make use of *f*_*s_x_*,*d*,*t*_ measurements over multiple time points, we integrate all abundance measurements to compute an area under the growth curve (***A**UC*) from timepoints 0 to *T* (the total number of pool generations measured). Here, frequencies between measured timepoints were linearly interpolated:

$$AUC_{s_{x},d}=k\int_{0}^{T}f_{t,s_{x},d}2^{t}dt$$

The estimated area under the growth rate can be similarly computed in terms of *f*_*s_x_*,*d*,0_ and the unknown growth rate *g*_*s_x_*,*d*_:

$${\hat{AUC}}_{s_{x},d}=k\int_{0}^{T}f_{0,s_{x},d}2^{{\hat{g}}_{s_{x},d}t}dt$$

Using this relationship between the estimated and observed ***A**UC*, we solved for ${\hat{g}}_{s_{x},d}$ in the following equation:

$$k\int_{0}^{T}f_{t,s_{x},d}2^{t}dt=k\int_{0}^{T}f_{0,s_{x},d}2^{{\hat{g}}_{s_{x},d}t}dt\;\;\int_{0}^{T}f_{s_{x},d,t}2^{t}dt=f_{s_{x},d,0}\frac{2^{{\hat{g}}_{s_{x},d}T}-1}{{\hat{g}}_{s_{x},d}T\;\log(2)}$$

Because $\int_{0}^{T}$ *f*_*s_x_*,*d*,*t*_2^*t*^*dt* and *f*_s_x_,*d*,0_ are both known, we numerically solve for the ${\hat{g}}_{s_{x},d}$ which best satisfies this relationship using the optimize() function in R (with a squared-error loss function). A minimum of −10 and maximum of 10 were used for the searched interval. Growth was estimated only for strains that were initially well-represented (average c_*o,s_x_*,*d*_>30, considering only available tags). The estimated *g*_*s_x_*,*d*_ represents the growth rate relative to the pool as a whole (i.e. a strain with *g*_*s_x_*,*d*_ = 1 perfectly keeps up with the pool). In practice, *g*_*s_x_*,*d*_ represents the average relative exponential growth rate from 0 to *T*. For example, a prolonged lag phase would effectively lower the average exponential growth rate. For linear regression and neural network training, the minimum $\hat{g}$ is set to 1e-10 to avoid numerical errors in the respective algorithms. To derive the resistance for each strain in each drug (*r*_*s_x_*,*d*_), the growth rate for each strain in a given drug *g*_*s_x_*,*d*_ is divided by the corresponding growth rate in the DMSO control (*g*_*s_x_*,*DMSO*_):

$$r_{s_{x},d}=\frac{g_{s_{x},d}}{g_{s_{x},DMSO}}$$

We note that experimental uncertainty in the collected generation times *t* can introduce some scale uncertainty in estimates of *r*_*s_x_*,*d*_, such that resistance estimates from the MAT**a** and MAT**α** pool may be highly correlated, but may differ in scale for some drugs (Figure S2). To adjust for any potential pool-of-origin effects in *r*_*s_x_*,*d*_ arising from merging the MAT**a** and MAT**α** populations, we use the line of best fit derived in Figure S2 to rescale *r*_*s_x_*,*d*_ estimates from the MAT**a** pool to match those from the MAT**α** pool before merging *r*_*s_x_*,*d*_ values from the two pools.

#### Generalized Linear Model of Genetic Effects

To model resistance (*r*) resulting from multiple genetic perturbations, we adopted the multiplicative model describing how genetic effects will combine in the absence of genetic interaction (Mani et al., 2008). When applied to two-gene effects, this model expresses the expected resistance of a double knockout strain (*x*Δ*y*Δ) in a given drug (${\hat{r}}_{x\Delta y\Delta ,d}$) as the product of the resistances of the component single-knockout strains:

(Equation 1) $${\hat{r}}_{x\Delta y\Delta ,d}=r_{x\Delta ,d}r_{y\Delta ,d}$$

To express this model in an additive form, we state this relationship as an exponentiated sum of the log-resistances of the single knockouts - log(r_*s_i_*,*d*_) = *I*_*s_i_*,*d*_, so that:

(Equation 2) $${\hat{r}}_{x\Delta y\Delta ,d}=\text{exp}(l_{x\Delta ,d}+l_{y\Delta ,d})$$

The simplest deviation from this model is a two-gene interaction. We define a two-gene interaction term (*ε*_*x*Δ*y*Δ,*d*_) as the log-ratio between the observed fitness, and the fitness expected by the multiplicative model of single-gene effects. This is used instead of the previously-described linear difference from a multiplicative estimate (*ε*_*x*Δ*y*Δ,*d*_ ≡ *r*_*x*Δ*y*Δ,*d*_ – *r*_*x*Δ,*d*_*r*_*y*Δ,*d*_ (St Onge et al., 2007)) to more readily generalize to higher-order effects:

(Equation 3) $$\varepsilon_{x\Delta y\Delta ,d}\equiv \log\left(\frac{r_{x\Delta y\Delta ,d}}{{\hat{r}}_{x\Delta y\Delta ,d}}\right)$$

Using this definition, the observed double mutant fitness can be expressed as the sum of the single-gene effects, and the interaction term from 2):

(Equation 4) $$r_{x\Delta y\Delta ,d}=\text{exp}\;(l_{x\Delta ,d}+l_{y\Delta ,d}+\varepsilon_{x\Delta y\Delta ,d})$$

To estimate the triple-mutant fitness expected from one- and two-gene effects in the absence of three-gene genetic interaction, all relevant single-knockout terms and two-gene interactions are added:

(Equation 5) $${\hat{r}}_{x\Delta y\Delta z\Delta ,d}=\text{exp}\;(l_{x\Delta ,d}+l_{y\Delta ,d}+l_{z\Delta ,d}+\varepsilon_{x\Delta y\Delta ,d}+\varepsilon_{x\Delta z\Delta ,d}+\varepsilon_{y\Delta z\Delta },d)$$

The three gene interaction term is the deviation from the above expectation from one- and two- gene effects:

(Equation 6) $$\varepsilon_{x\Delta y\Delta z\Delta ,d}\equiv \log\left(\frac{r_{x\Delta y\Delta z\Delta ,d}}{{\hat{r}}_{x\Delta y\Delta z\Delta ,d}}\right)$$

This definition can be extended analogously for interactions of arbitrary complexity, with *ε* terms denoting interactions between the corresponding knockouts. Specifically, we fit a generalized linear model to predict the fitness of each strain to a drug, given its knockout genotype (***G***Δ). ***G***Δ is a subset of 16 ABC transporter knockouts {***A****BC*1_Δ_…***A**BC*16_Δ_}:

(Equation 7) $${\hat{r}}_{s_{x}|G\Delta \;\subseteq \;\{ABC1_{\Delta }\ldots \;ABC16_{\Delta }\},d}=\text{exp}\left(\sum_{i\in G\Delta }l_{i,d}+\sum_{j\subseteq G\Delta }\varepsilon_{j,d}+c_{d}\right)$$

Here, the *l*_*i*,*d*_ coefficients are single-knockout resistance effects for a drug, while the *ε*_*j*,*d*_ coefficients are interactions between two or more genes (i.e. subsets of two or more elements from ***G***Δ). *c*_*d*_ is an offset term in each drug, and defines the predicted resistance of a strain with no modeled genetic effects (i.e. a wild-type like strain). We further extended this model to capture potential biases in each drug associated with the plate-of-origin, assigning a single ‘bias’ (*b*) coefficient to each strain, given its origin from one of 30 plates (*b_p_*):

(Equation 8) $${\hat{r}}_{s_{x}|G\Delta \;\subseteq \;\{ABC1_{\Delta }\ldots \;ABC16_{\Delta }\},\;p,d}=\text{exp}\left(\sum_{i\in G\Delta }l_{i,d}+\sum_{j\subseteq G\Delta }\varepsilon_{j,d}+c_{d}+b_{p}\right)$$

To train this model, ***G***Δ is encoded as a set of 16 binary variables, where 0 represents a wild-type and 1 represents a knockout at a given gene. Therefore, to predict phenotype from ***G***Δ, the relevant *l*_*i*_ coefficients are added only if the corresponding gene *i* is knocked out, and the *ε_j_* coefficients are added only if all the genes in subset *j* are knocked out. For each drug, we fit this model using the glm() function in R, with *ε* terms to a chosen level of complexity.

#### Defining a Neural Network System Model

To model the dependence of drug efflux on transporter genotype, we defined a neural network that learns about influence between transporters, and the relationship between transporter activities and drug resistance. We structured the neural network model (Figure 5A) to have three layers: 1) an input layer encoding the binary genotype ***G*** for each of the 16 targeted transporters (***G***); 2) a middle ‘hidden’ layer encoding values (***A***; ranging from 0 to 1) that estimate the activity of each of the 16 transporters (***A***); and 3) an output layer that quantitatively describes resistance to each of 16 drugs (***R***; ranging from 0 to 1). To represent regulatory influence relationships between transporters, the links between genotype and activity layers have (initially unknown) ‘influence’ weights (***I***), with positive weights where gene presence increases activity and negative weights where gene presence decreases activity. The links between activity and resistance layers have (initially unknown) non-negative ‘efflux’ weights (***E***) that capture the extent to which each transporter can catalyze the efflux (or otherwise reduce the activity) of each drug. The model also allowed for offset terms in both ***A*** and ***R***.

First, we rescaled all resistance measures for each drug to be between 0 – 1 by dividing with the maximum observed resistance (and setting a minimum of 1e-10):

$$r_{norm_{d}}\equiv \frac{r_{d}}{\max(r_{d})}$$

We then model a sigmoidal relationship between drug concentration and normalized resistance:

$${\hat{r}}_{norm_{d}}=\frac{1}{1+e^{k[d]-a}}$$

Here [*d*] is the concentration of a given drug, and *k*, *a* are unknown constants which define the dose-response curve (such that $\frac{a}{k}$ yields the expected IC50). This equation defines a baseline dose-response curve for a drug in a strain with no ABC transporters. From this baseline, the subset of 16 ABC transporters present in a strain *i*(***G***_*i*_ ⊆ {***A**BC*1^+^ … ***A**BC*16^+^}) act to additively lower the effective concentration of a drug (for example, by efflux out of the cell). Thus, each transporter is given a non-negative clearance coefficient *C* for each drug, such that:

$${\hat{r}}_{norm_{G_{i},d}}=\frac{1}{1+e^{k[d]-a-\sum C_{i},d}}$$

Importantly, a dose-response curve in this form can be expressed as the activation of a sigmoid neuron, where *k*[*d*]–a is collapsed into a single bias term for each drug (*B*_*d*_), and *C*_*i*,*d*_ are the weights learned as inputs to this neuron from the ABC transporters. In this model, each transporter must act to lower effective drug concentration, so we constrain *C*_*i*,*d*_ to be non-negative.

In addition, we model influence between ABC transporters. To do this, we first decompose the clearance coefficient of each ABC transporter (*C*) as the degree of transporter activity (***A***, a value between 0 and 1), and its potential efflux capacity (***E***), so that *C* = ***AE***. We modeled a common set of influence relationships across drugs, so that transporter activity is modeled as being dependent on ***G**_i_*, but not the drug *d* (***A**_i_*). Therefore, *C*_*i*,*d*_ = ***A**_i_**E***_*i*,*d*_, such that for each drug:

$${\hat{r}}_{norm_{G_{i},d}}=\frac{1}{1+e^{-\sum A_{i}E_{i,d}-B_{d}}}$$

***A**_i_* allows our model to capture that the activity of each ABC transporter can be additively influenced by other transporters. Each activation value in a strain (***A***_*j*∈***G**_i_*_) is computed by the influences from other ABC transporters (***l**_k_*):

$$A_{j\in G_{i}}=f\left(\sum_{k\in G_{i},j\neq k}I_{k}\right)$$

While the form of the function computing each ***A***_*j*∈***G**_i_*_ is itself unknown, here we also modeled it as a sigmoidal function for simplicity:

$$A_{j\in G_{i}}=\frac{1}{1+e^{-\sum I_{k}-B_{A_{j}}}}$$

Here, *B*_***A**_j_*_ is an offset term which defines the baseline activation of each transporter in the absence of influence connections.

#### Learning Neural Network Parameters

To create the above model and learn the ***I*** and ***E*** parameters from our data, we used the keras library in R to construct a neural network of the appropriate form.

We first provided the genotype of each strain as the input to the neural network by encoding ***G*** in binary form. That is, we created an input layer of length 16, where each input value will be either 1 (denoting ABC transporter presence), or 0 (denoting a knockout) for each of {***A**BC*1…***A**BC*16}.

We then created a second layer of length 16, where the weights from the input genotype (***G***) layer to the ‘activity’ (***A***) layer encode the influence weights (***I***) from transporter *i* to transporter *j*(***I***_*i*,*j*_). Learned ***I***_*i*,*j*_ weights are used (along with offset terms *B_**A**_*) to compute the activity state for each transporter (***A**_j_*), given **G**. Specifically, we created a second activity layer of length 16, and connected each ***G***_*i*_ to each ***A**_j_* with a sigmoid activation function, omitting self-self connections. The neural network model multiplies these learned ***I***_*i*,*j*_ weights by ***G***, such that all outgoing influences from transporter *i* are set to 0 if it is knocked out. Similarly, we set the activation state of each transporter in the second layer ***A**_j_* to 0 if it is knocked out To achieve this, we multiplied ***A*** element-wise by ***G*** using the layer_multiply() function. In addition to enforcing the expected behavior that a transporter should not provide efflux activity if it has been knocked out, this associates each node in the activity layer with a specific gene, making more interpretable what might otherwise be a ‘black box’ hidden layer.

To model the efflux weights (***E***) for each transporter-drug pair ***E***_*j*,*d*_, we connected the activity of each transporter in the ***A*** layer (***A**_j_*) with each of 16 drugs in the third resistance (***R***) layer via links representing sigmoid activation functions. Each ***R*** node models the normalized resistance to each compound (${\hat{r}}_{norm_{G,d}}$) by multiplying ***A**_j_* with the learned efflux weights ***E***_*j,d*_. ***A***×***E*** computes the clearance coefficients for each drug-transporter pair (*C*_*j*,*d*_), which is used along with the learned offset terms for each drug (*B*_*d*_) to compute ***R*** from ***G***. To learn non-negative parameters for ***E***, we used the kernel_constraint argument in keras.

To learn a sparse predictive model, we added L1 regularization (with coefficient *λ*) to the ***I***_*i*,*j*_ and *B*_***A***_ weights (both used to compute ***A***). This avoids learning extraneous weights which do not affect phenotypic predictions. For example, regularization on ***I***_*i*,*j*_ weights penalizes influence relationships between transporters that do not have any non-zero ***E***_*j*,*d*_ weights. Similarly, regularization on *B*_***A***_ avoids setting the baseline activation for a transporter near 0 by effectively setting a prior to an ***A*** (so that it remains close to 0.5 unless otherwise supported by data). This prior on ***A*** indirectly penalizes ***E***_*j*,*d*_ weights that do not affect the resulting clearance coefficients (because *C* = ***A*** × ***E***, ***E*** can vary freely if ***A*** ≈ 0). While more complex regularization schemes can potentially impose three separate regularization weights for ***I***_*i*,*j*_, *B_**A**_*, and ***E***_*j*,*d*_, we found that using a single *λ*coefficient for regularizing both ***I***_*i*,*j*_ and *B_j_*, without any further regularization to ***E***_*j*,*d*_ was sufficient for learning a sparse predictive model. Regularization was added to the model using the kernel_regularizer() argument.

The neural network model was compiled with the mean-squared error (‘mse’) loss function, using the adam optimizer with a learning rate of 0.05 when training using data for all drugs. When neural network model training was performed using only data from a single drug, e.g. valinomycin or fluconazole (Figures 5D, 6E, S5E, and S6E), we found empirically that setting the learning rate to 0.01 lowered variance in parameter values between different training runs. Training was performed for 10,000 epochs, using a batch size of 30%, and a 10% split between training and validation (validation_split = 0.1).

#### Extensions to the Neural Network

Based on poor fit of our original model to the valinomycin data, and based on the previous observation that the ABC16 strain is more resistant to valinomycin than wild-type (Suzuki et al., 2011), we hypothesized that some subset of ABC transporters act to influence an unknown valinomycin resistance factor. To extend the neural network for valinomycin to capture this scenario (Figure 5D), we added a single ‘always-present’ factor in ***G***. More specifically, we added one extra variable to ***G*** and set its value to 1 for each strain.

To train a neural network with both direct and indirect influence connections using fluconazole resistance data (Figure 6E), we first restricted ***G*** in the first layer to encode only the presence of the frequently-associated transporters *PDR5*, *SNQ2*, *YBT1*, *YCF1*, and *YOR1*, and restricted ***A*** to encode only the efflux activity of *PDR5* (i.e. ***A*** = ***A***_*PD**R***5_). To model a hidden factor providing additional indirect connections between ***G*** and ***A***_*PD**R***5_, we added an additional ***A***′ layer consisting of a single $A_{\mathrm{PD}R5}^{\prime }$ node. $A_{\mathrm{PD}R5}^{\prime }$ computes its value using a set of indirect connections (***I***_2_) from ***G*** (from all transporters except *PDR5*), and then connects to ***A***_*PD**R***5_. Thus, influence from ***G*** to ***A***_*PD**R***5_ can be computed using the direct influence links in the original model (***I***_1,*PD**R***5_), as well as the indirect ***I***_2,*PD**R***5_ influences integrated by $A_{\mathrm{PD}R5}^{\prime }$. As with the original model, additional influence connections, as well as the bias on ***A***′, were subject to L1 regularization with rate *λ*.

#### Growth Profiling for Individual Strains

Individual strains with 32 knockout combinations at *PDR5, SNQ2, YBT1, YCF1*, and *YOR1* were each grown in fluconazole at concentrations of 1.9, 3.9, 7.8, 15.6, 23.4, 31.2, 35 and 40μM. Each genotype was grown an average of 2.7 times (range 1 – 4) in each concentration. For each growth experiment, a culture was started at 2% DMSO at the same time to act as a solvent control. Each culture was started at an initial cell concentration of 0.0625 OD600. OD600 was measured every 10 minutes using a Tecan plate reader for a minimum of 20 hours.

#### MYTH Testing of Protein-Protein Interactions

MYTH bait/prey generation and testing were carried out as previously described (Snider et al., 2013). Briefly, *PDR5*, *YOR1*, and *SNQ2* iMYTH baits were generated by stable, in-frame, genomic integration of a Cub-LexA-VP16 tag (obtained from an L2 cassette) at the 3’ end of each gene. An integrated MYTH-tagged artificial bait was used as a negative bait control. *PDR5*-NubI and *PDR5*-NubG prey plasmid constructs were prepared using a pPR3N MYTH-tagging vector. Previously-generated Ost1p-NubG and Ost1p-NubI prey plasmid constructs were used as negative and positive interaction controls, respectively. Bait-prey combinations were obtained by chemical transformation of prey plasmid into each bait strain, followed by selection on SD –Trp (SD –W) media. Colonies of transformed strains were regrown on solid medium for 5 days using SD–W, SD–Trp–Ade–His (SD–WAH), SD–WAH +25μM fluconazole + 0.05% DMSO, SD –WAH +50μM fluconazole + 0.05% DMSO, and SD –WAH + 0.05% DMSO.

#### PCA Testing of Protein-Protein Interactions

*PDR5*, *YOR1*, and *SNQ2* MAT**a** (mDHFR-F[1,2]-NatMX fusions) and MAT**α** (mDHFR-F[3]-HphMX fusions) PCA strains were obtained from a previous genome-wide screen (Tarassov et al., 2008). Additional strains acting as positive and negative interaction controls were also obtained from this screen (Zip-F[1,2]/Zip-F[3] and Link-F[1,2]/Link-F[3], respectively). Strains were individually mated and diploids were selected on solid YPD supplemented with Hygromycin B and Nourseothricin (YPD +Hyg +Nat). Diploid strains were spotted on solid YPD +Hyg +Nat supplemented with either 2% DMSO, 2% DMSO + 200 μg/mL methotrexate, or 2% DMSO + 200 μg/mL methotrexate + 46.8μM fluconazole. Strains were grown for 72 hours at 30°C.

#### Quantitative RT-PCR

RNA was extracted from cultures growing exponentially in 23.43μM fluconazole using the QIAGEN RNeasy^®^ kit. 1μg of isolate was treated with DNAse and analyzed using an Agilent Bioanalyzer to quantify nucleic acid concentration and verify purity. cDNA synthesis was performed using a combination of oligo-DT and random hexamer primers using the Thermo Scientific™ Maxima™ H Minus First Strand cDNA Synthesis Kit. qPCR on these samples was then performed using a Bioline SensiFAST™ SYBR No-ROX qPCR kit and Ct values were quantified using a CFX machine. cDNA synthesis and qPCR was performed for *PDR5* and *UBC6* (which acted as loading control).

### QUANTIFICATION AND STATISTICAL ANALYSIS

#### Genetic Interaction Significance Testing

To perform the marginal association in Figure 2A, we fit the generalized linear model of genetic effects with no *ε_j_* terms, and performed stepwise feature elimination (eliminating the gene with the highest *p*-value at each step) until all included terms had a significance level of *p*≤0.05/16. Linear model term significance was tested using the Type III Sums of Squares ANOVA implementation given in the car package in R. The same modeling procedure was used to perform the marginal association in Figure S2A, substituting *g* for *r*.

To generalize the marginal association approach for training models containing *ε* terms of up to *n*-way complexity (5-way in this study), we used a ‘stepwise expansion’ method. First, we use the marginal association procedure to initialize the model at *n* = 1. Then, *n* is incremented by 1, and all possible *n*-way interactions between the genes contained in the existing (i.e. *n* – 1) model are added as additional *ε_j_* features. Each term in this proposed *n*-way model is tested for significance using Type III Sums of Squares ANOVA, those with *p* ≤ 0.05 are discarded, and the model is updated. This stepwise expansion procedure is repeated until either *n* reaches 5, or the number of genes in the *n* – 1 model is less than *n* (i.e. there are no more interaction terms to search for). After the stepwise expansion procedure is finished, the remaining terms are more rigorously tested for statistical significance by performing stepwise feature elimination (as in the marginal association procedure) until all included terms have a significance level of *p*≤0.05/*k*, where *k* is the number of all possible 1-5 gene combinations amongst the marginally-associated genes.

#### Further Neural Network Weight Regularization

All neural networks were trained 10 times, varying the initial parameter values and the stochastically sampled gradients in each run. The weights to the final model were set to the mean weights learned from these 10 training iterations. In addition, standard deviation between these 10 iterations was calculated for each parameter, and was used to compute an absolute parameter Z score:

$$|Z_{param}|=\frac{|\mu_{param}|}{\sigma_{param}}$$

Given the non-deterministic nature of the algorithm, we wanted to ensure that non-zero parameters are not a result of stochastic variability, and therefore non-zero weights with ∣*Z*_*param*_∣ < 4 were set to 0.

We further examined each non-zero weight to assess its predictive value. First, we computed the vector of squared residuals in the initial model over *i* strains and *j* drugs, given the set of *k* initial non-zero weights *W*_{1–*k*}_:

(εinitial)2=((rnormG{1…i},d{1…i}−r^normG{1…i},d{1…i}∣W{1…k})2

Then, for each *I*∈ {1 …*k*}, we set *W*_*I*_ to 0, and computed the squared residuals in the proposed reduced model:

(εreduced)2=((rnormG{1…i},d{1…j}−r^normG{1…i},d{1…j}∣W{1…(l−1),(l+1)…k})2

Considering only data where setting *W*_*l*_ to 0 made a predictive difference (*ε*_*initial*_ ≠ *ε*_*reduced*_ at a numerical tolerance of 10^−4^), we then computed the paired Mann-Whitney U statistic between (*ε*_*initial*_)^2^ and (*ε*_*reduced*_)^2^ to derive a *p*-value for the degree of squared-error reduced by *l*, and keep all features with *p*<0.05/*k* in the final model.

We searched for an appropriate regularization rate (*λ*) by performing the above training, merging, and pruning procedure using a range of rates from 10^−6^ to 10^−1^. We first searched 13 intervals between 10^−6^ to 10^0^(Figure S5A). We observed high mean-squared error (MSE) and a lack of reproducible parameters at regularization rates below ~ 10^−4.5^ and a smaller increase in MSE around ~ 10^−3^, we searched another 11 intervals between 10^−4^ to 10^−3^ (Figure S5A). We chose a regularization rate of 5×10^−4^ for the model in Figure 5B, as any rate higher than this resulted in a jump in MSE in both the MAT**a** and MAT**α** pools, while lowering this rate did not have a clear impact on MSE, but increased the number of non-zero parameters (Figure S5A).

To regularize weights for the neural network in Figure 6E, we initially employed the testing procedure described above, but observed occasional convergence on a set of parameters with a high mean-squared error, even at high regularization rates. We therefore modified the model merging procedure to use the median weights between 10 runs rather than the mean, and discontinued use of the ∣*Z*_*param*_∣ filter. Furthermore, we searched for a different *λ* for this network because training with *λ* = 5×10^−4^ resulted in a similar model as the two-layer network (data not shown). Therefore, we performed a separate ‘three-layer *λ*’ search for this network, searching 13 intervals between 10^−6^ to 10^0^ (Figure S6D). For three-layer training, we found that *λ*> 10^−5^negatively impacts MSE (Figure S6D), and therefore used a less-restrictive *λ* = 10^−5^ to train the three-layer network in Figure 5B. The predictive value of the learned weights was subject to the same statistical significance test as for the two-layer network.

#### Analysis of Liquid Growth Data

To calculate resistance, we divided the OD measured in the drug by the OD measured in the solvent at the time which the culture first saturated in the solvent. To automatically determine a saturation timepoint, we took the second derivative of the growth curve (using a window size of 4 tecan measurements to calculate the first derivative) and determined the time which it is maximized. Automatically determined saturation times were checked visually. Multiple replicates were averaged to yield the values in Figure S6A. To determine the fitted IC50 values in Figure 6B, averaged resistance values were linearly interpolated between measured concentrations.

#### Quantitative RT-PCR Analysis

Relative expression of *PDR5* in all strains was calculated as 2^*C_q_UBC*6–*C_q_PD**R***5^. For each strain, *C*_*q*_ values for the cDNA samples were quantified multiple times to assess technical variability (*C*_*q*_*PD**R***5 was measured in triplicate, and *C*_*q*_*UBC*6 was measured in triplicate), and these multiple measurements were averaged before calculating relative expression. qRT-PCR was performed for three individual cultures of each strain in each genetic background. RY0566 was used as the wildtype.

### DATA AND CODE AVAILABILITY

R scripts used to perform computational analyses are available at https://github.com/a3cel2/xga. High-throughput sequencing reads generated for *en masse* genotyping and BarSeq are available at the NCBI Sequence Reads Archive, with accession SRA: PRJNA535622.

## Supplementary Material

1

2

3

4

5

6

7

## Figures and Tables

**Table:** KEY RESOURCES TABLE

Reagent or Resource	Source	Identifier
Chemicals, Peptides, and Recombinant Proteins		
fluconazole	Sigma-Aldrich	F8929
ketoconazole	Sigma-Aldrich	K1003
miconazole	Sigma-Aldrich	1443409
itraconazole	Sigma-Aldrich	I6657
beauvericin	Sigma-Aldrich	B7510
tamoxifen	Sigma-Aldrich	T5648
benomyl	Sigma-Aldrich	45339
cycloheximide	Sigma-Aldrich	C1988
methotrexate	Sigma-Aldrich	M9929
camptothecin	Sigma-Aldrich	C9911
cisplatin	Sigma-Aldrich	P4394
bisantrene	Sigma-Aldrich	B4563
mitoxantrone	Sigma-Aldrich	6545
colchicine	Sigma-Aldrich	9754
imatinib	Sigma-Aldrich	270784
valinomycin	Sigma-Aldrich	V3639
Deposited Data		
High-throughput sequencing reads generated for *en masse* genotyping and BarSeq	This Paper, SRA	SRA: PRJNA535622
Experimental Models: Organisms/Strains		
RY0622	Suzuki et al., 2011	N/A
RY0146	Suzuki et al., 2011	N/A
RY0566	Suzuki et al., 2011	N/A
RY0148	Suzuki et al., 2011	N/A
Barcoded RY0148 pool	This paper	N/A
Oligonuclides		
All DNA primers used, see Data S1	This paper	Data S1
Recombinant DNA		
Plasmid: pSH47	Euroscarf	P30119
Plasmid: pIS420	Euroscarf	P30575
Software and Algorithms		
Analysis pipeline (written in R)	This paper	https://github.com/a3cel2/xga
R 3.4.3	R Core Team	https://www.r-project.org/

## Highlights

- Describes XGA, a strategy for analyzing highly combinatorial genetic perturbations
- XGA of 16 ABC transporters revealed many high-order genetic interactions
- “Visible neural network” modeling yielded intuitive transporter influence models
- Results motivate XGA as a general strategy to model complex traits
