# Supplementary material for: Highly Combinatorial Genetic Interaction Analysis Reveals a Multi-Drug Transporter Influence Network
Source: Cell Syst. Author manuscript; Available in PMC 2021 Jan 22. (PMC6989212; doi:10.1016/j.cels.2019.09.009)
Supplement: 1 [file NIHMS1545864-supplement-1.pdf]

Figure S1

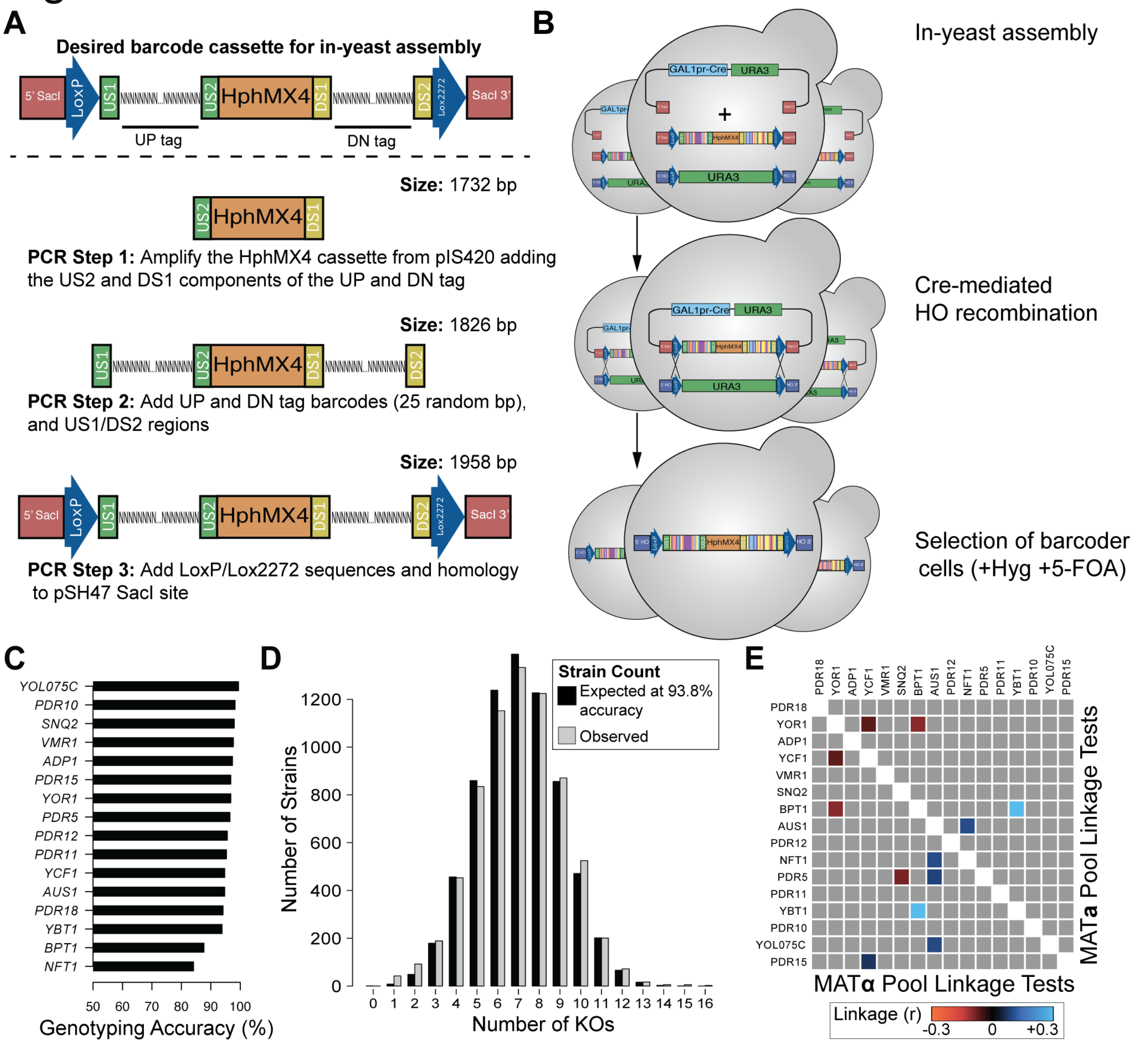

Figure S1. Barcoder Pool Creation and Genotyping Quality, Related to Figure 1

(A) Three PCR reactions create the desired barcode cassette for use with in-yeast assembly. See STAR Methods for details.

(B) Transforming a pool of barcoder parents. The barcode cassette was co-transformed with a SacI-digested pSH47 into a modified RY0148 strain containing a *loxP-URA3-lox2272* site at the *HO* locus. Cre induction enables recombination and replacement of *URA3* with the barcoder pool cassette. Loss of *URA3* is then selected by subsequent growth in 5-FOA.

(C) Expected genotyping accuracy at the 16 ABC transporters surveyed. Accuracy was estimated by evaluating the performance of the RCP-PCR genotyping protocol on a set of individually-genotyped 'gold standard' strains (See STAR Methods, Data S2).

(D) Distribution of knockouts in the combined MAT**a** and MATα pools. The observed number of strains with a given number of knockouts are indicated in gray. The expected number of strains with a given number of knockouts at 93.8% genotyping accuracy are indicated in black.

(E) Tests of gene linkage within the MAT**a** pools (upper triangle) and MATα pools (lower triangle). The Pearson correlation coefficient of the corresponding genotype pairs are indicated on the right. Pairs without significant correlation (Multiple-testing adjusted p value ≥ 0.05) are shaded in gray. Three pairs of unlinked genes – *YOR1-YCF1*, *YOR1-BPT1*, and *SNQ2-PDR5* – exhibited weak but significant negative correlation in the appearance of KO genotypes (-0.04 ≥ r ≥ -0.08). This effect may have arisen via negative genetic interactions conferring lower growth for the corresponding double-knockout genotypes during the sporulation, haploid selection, or automated colony picking steps.

Figure S2

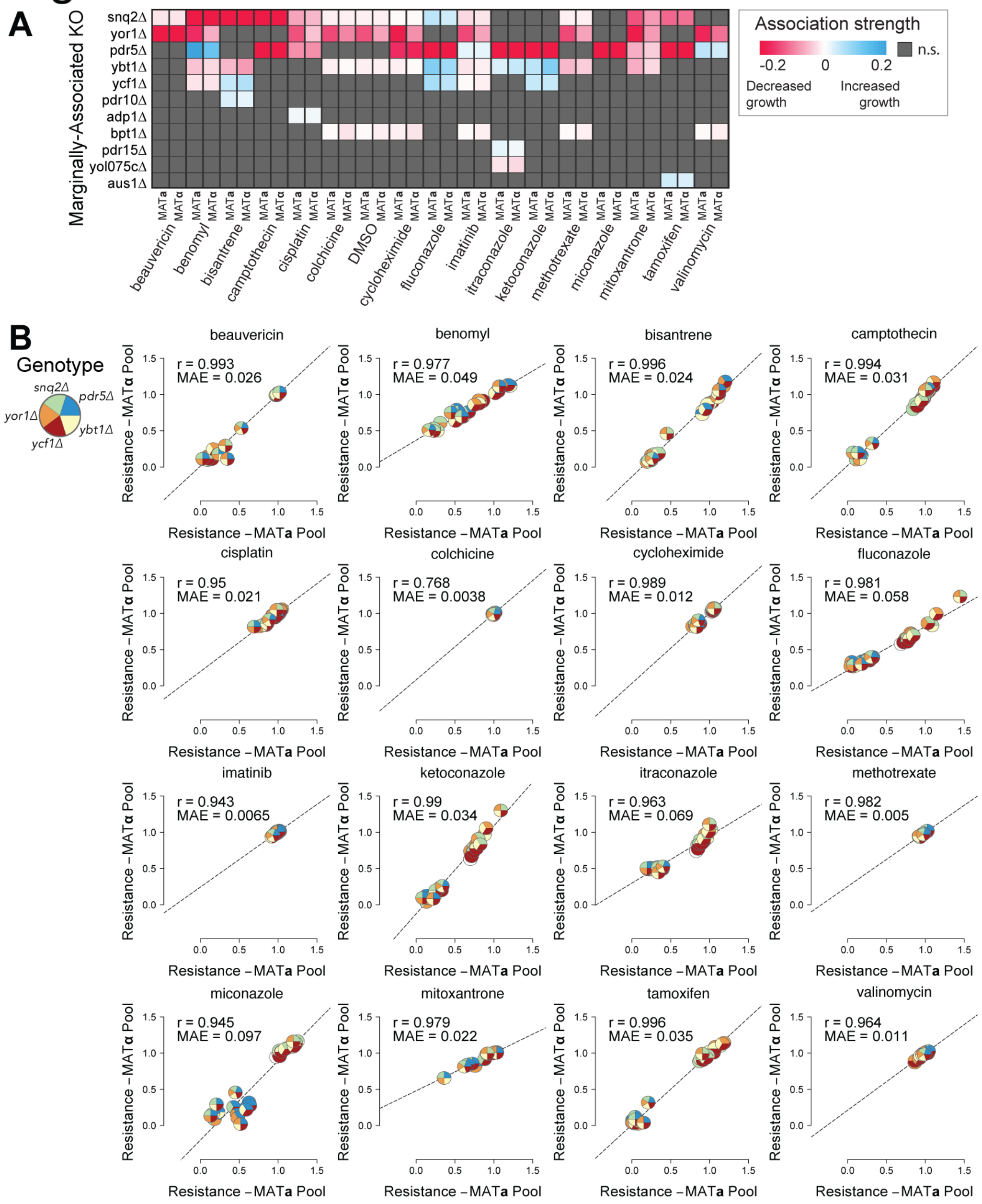

Figure S2. Marginal Growth Effects and all Five-Gene Averaged Resistance Profiles, Related to Figure 2

(A) As in Figure 2A, showing single-gene marginal effects that mediate growth rather than drug resistance.

(B) As in Figure 2B, comparison of five-gene averaged resistance profiles between biological replicates (MAT**a** and MATα pools) for each drug. Pearson correlation (*r*) and mean absolute error (MAE) between biological replicates are shown for each drug. MAE was estimated as the residual term in the line of best fit.

Figure S3

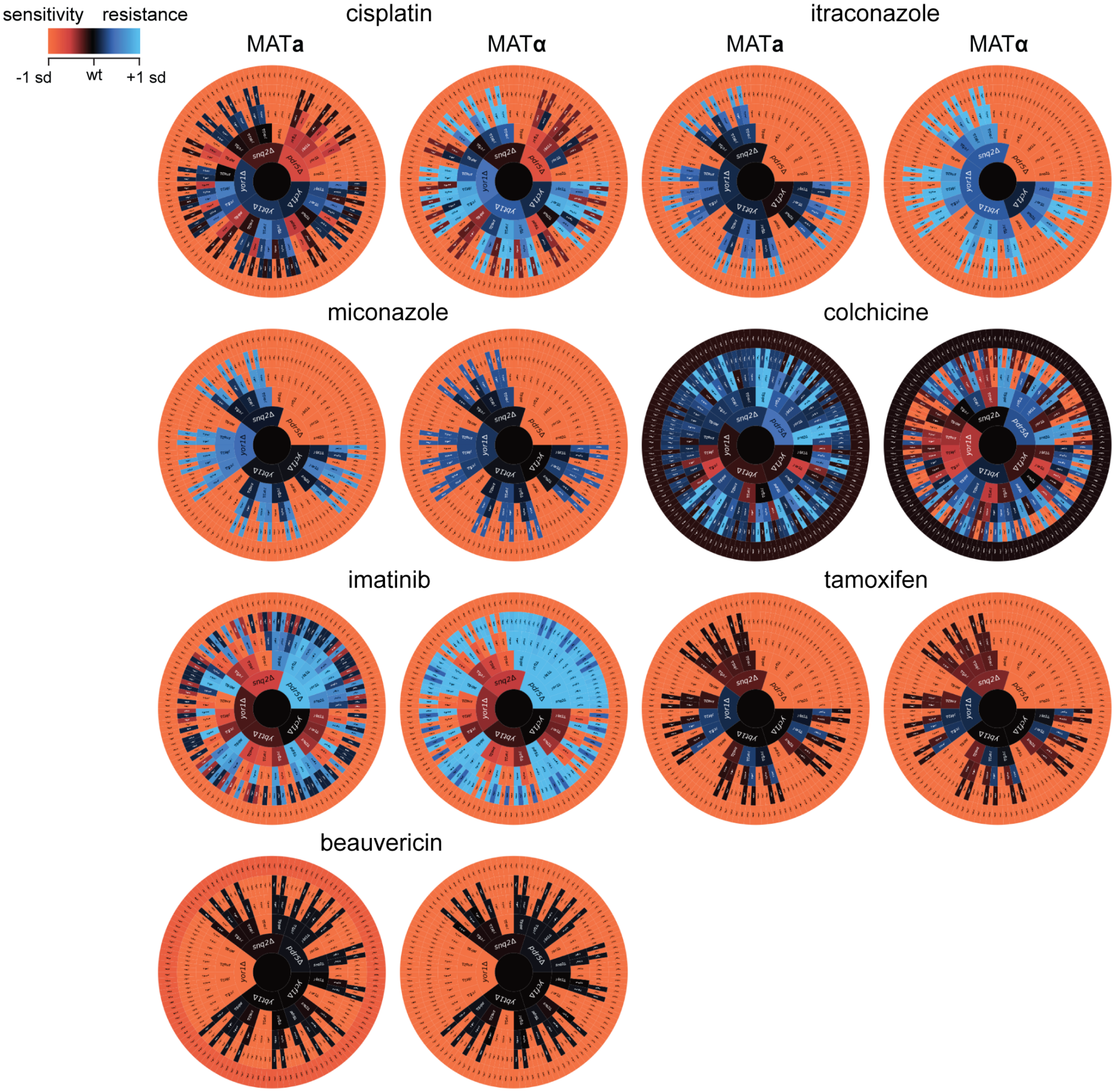

Figure S3. XGA Wheels for Additional Drugs, Related to Figure 2
As in Figure 2D, showing XGA wheels for seven additional drugs

Figure S4

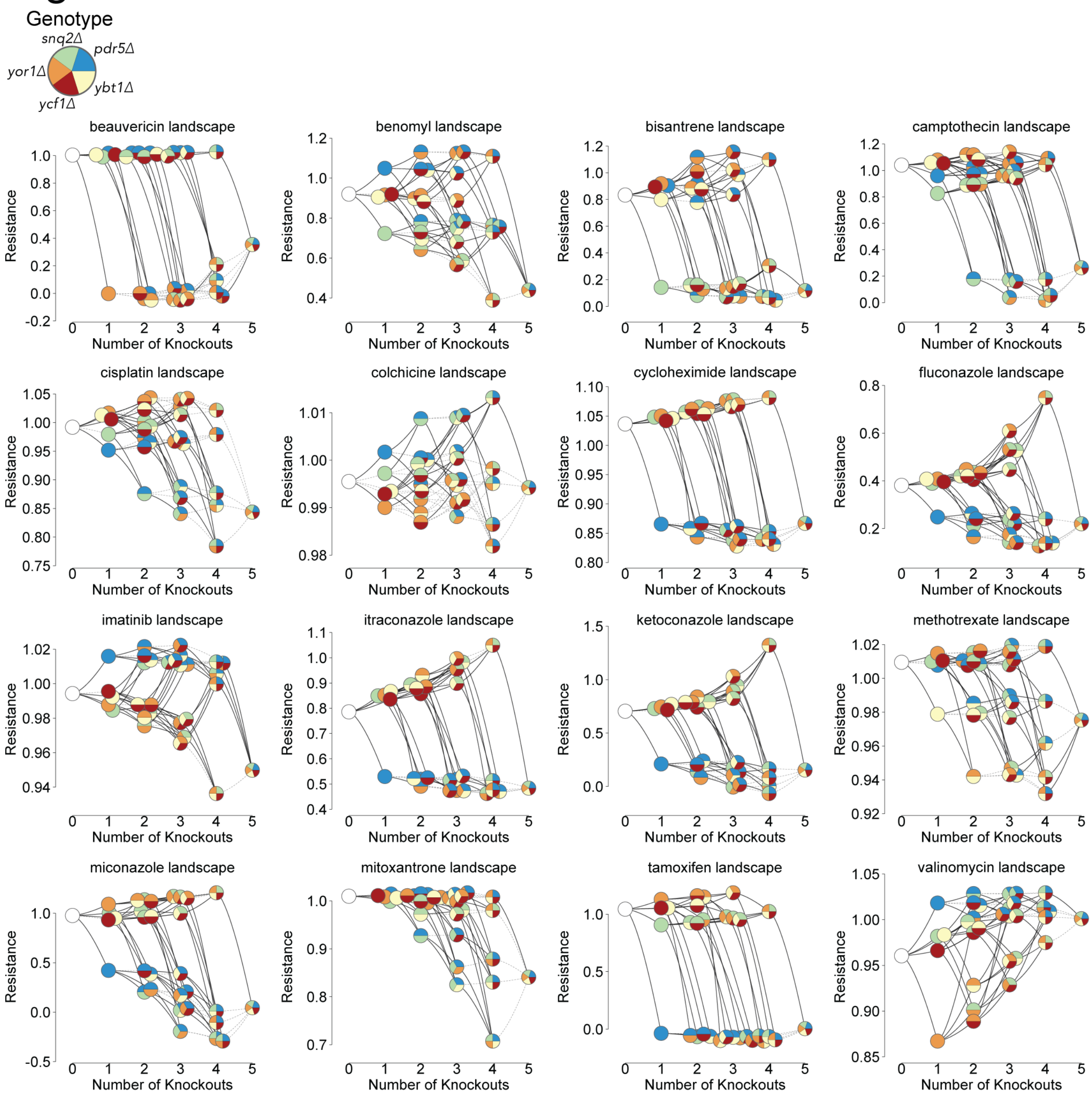

Figure S4. Resistance Landscapes for all Drugs, Related to Figure 3
As in Figure 3, showing all 16 drugs

Figure S5

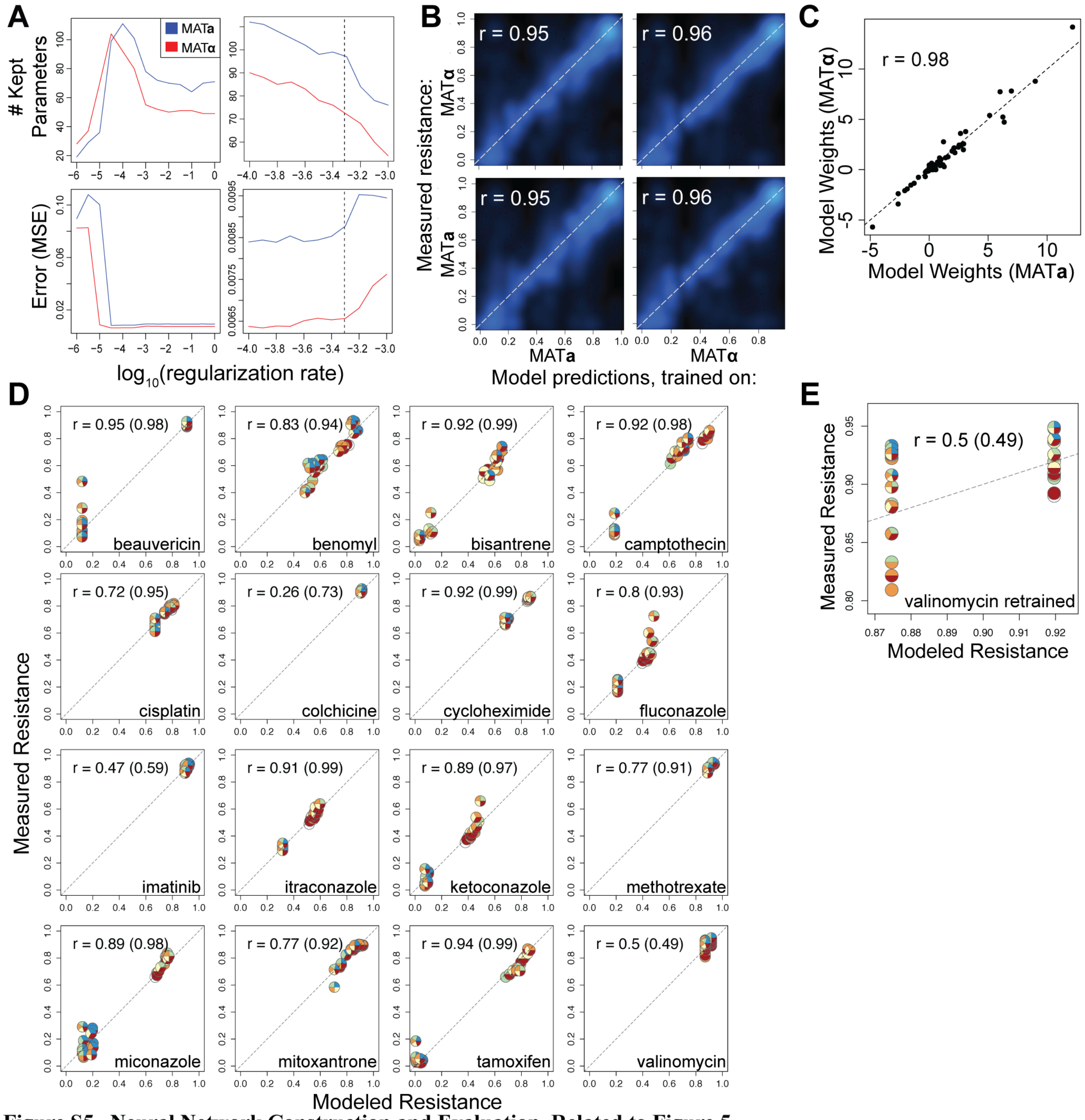

Figure S5. Neural Network Construction and Evaluation, Related to Figure 5

(A) Evaluating the number of kept network parameters (filtered for reproducibility and predictive support; top panels) and overall mean-squared error (MSE; bottom panels) as a function of the L1 regularization rate λ. 13 intervals are plotted from 10^{-6} to 10^{0} (left), and 11 intervals are plotted from 10^{-4} to 10^{-3} (right).

(B) As in Figure 5C, results are shown when the network is trained on either the MAT**a** or MATα population, and then tested on either the MAT**a** or MATα population.

(C) Comparing the learned network weights when the network is trained on either the MAT**a** or MATα population separately.

(D) As in Figure 5D, showing neural network performance for each drug. Here, correlation values are shown considering all resistance measurements, and those considering only only five-gene groups are in parentheses.

(E) As in Figure S5D, performance is shown for the original network (Figure 5A) re-trained using only valinomycin data. No substantial improvement over the original neural network is evident.

Figure S6

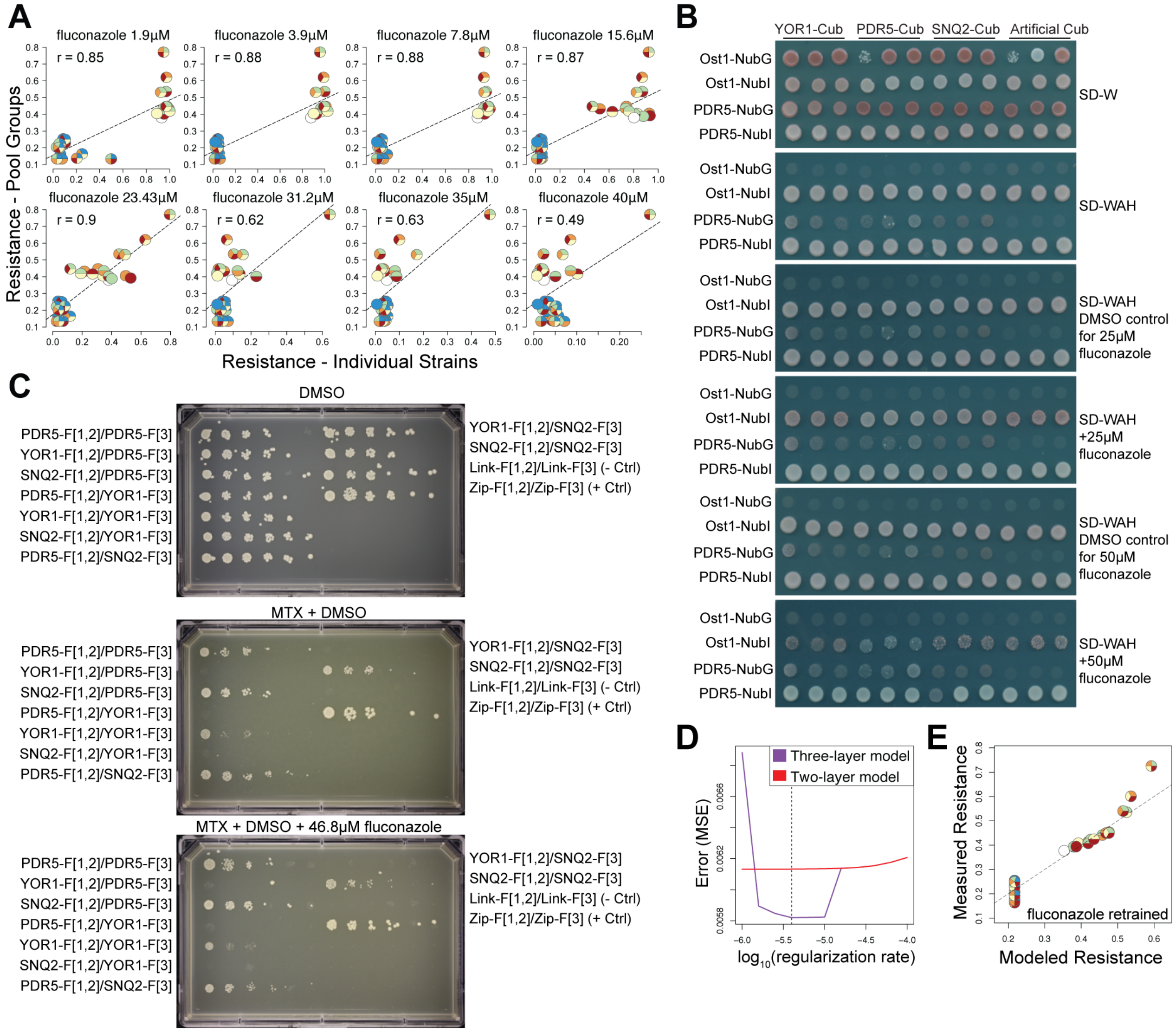

Figure S6. Additional Data for Deciphering a Complex Fluconazole Resistance Trait, Related to Figure 6

(A) Fluconazole resistance of individual strains containing 32 knockout combinations of *pdr5*Δ, *snq2*Δ, *ybt1*Δ, *ycf1*Δ, and *yor1*Δ are compared to the normalized grouped resistance profiles from the competitively grown pool data (Figure 6A) at the indicated concentrations. The pool was grown at 23.4μM.

(B) Measuring protein-protein interactions between Pdr5, Snq2, and Yor1 using mDHFR PCA. MAT**a** (mDHFR-F[1,2]-NatMX fusions) and MATα (mDHFR-F[3]-HphMX fusions) PCA strains were mated to obtain the indicated diploids. MTX selects for protein-protein interactions by reconstitution of mDHFR from F[1,2] and F[3] fragments.

(C) Measuring protein-protein interactions of Pdr5 with Snq2 and Yor1 using MYTH. NubG-*PDR5*, NubI-*PDR5*, Ost1-NubG, and Ost1-NubI strains were each transformed with plasmids containing clones of *PDR5*, *YOR1*, *SNQ2*, or an artificial bait fused to Cub. NubI fusions are expected to spontaneously reconstitute ubiquitin with Cub, while NubG fusions require a protein-protein interaction for reconstitution. Ost1 is not expected to interact with any baits tested. Condition abbreviations: SD –Trp (SD –W), SD –Trp–Ade–His (SD-WAH). SD –WAH conditions select for reconstitution of ubiquitin.

(D) As in Figure S5A, showing the mean-squared error (MSE) of both the original (two-layer) and the extended (three-layer) fluconazole resistance model as a function of the regularization rate λ. 13 intervals are plotted from 10^{-6} to 10^{-4}. The model with the lowest MSE ($\lambda = 10^{-5.4}$) was used in Figure 6E.

(E) As in Figure 5D, showing the original neural network trained only on fluconazole data. No substantial predictive improvement is evident for resistant multi-knockout groups when using the model with the lowest MSE ($\lambda = 10^{-5.8}$)
